# FGF19 induces the cell cycle arrest at G2-phase in chondrocytes

**DOI:** 10.1038/s41420-023-01543-6

**Published:** 2023-07-15

**Authors:** Hao Chen, Jiazhou Li, Caixia Pi, Daimo Guo, Demao Zhang, Xuedong Zhou, Jing Xie

**Affiliations:** 1 State Key Laboratory of Oral Diseases, West China Hospital of Stomatology, Sichuan University, 610041 Chengdu, China; 2 National Clinical Research Center for Oral Diseases, West China Hospital of Stomatology, Sichuan University, 610041 Chengdu, China; 3 Department of Cariology and Endodontics, West China Hospital of Stomatology, Sichuan University, 610041 Chengdu, China

**Keywords:** Cell-cycle exit, Kinases

## Abstract

Fibroblast growth factor 19 (FGF19) has appeared as a new possible avenue in the treatment of skeletal metabolic disorders. However, the role of FGF19 on cell cycle progression in skeletal system is poorly understood. Here we demonstrated that FGF19 had the ability to reduce the proliferation of chondrocytes and cause cell cycle G2 phase arrest through its interaction with β-Klotho (KLB), an important accessory protein that helps FGF19 link to its receptor. FGF19-mediated cell cycle arrest by regulating the expressions of cdk1/cylinb1, chk1 and gadd45a. We then confirmed that the binding of FGF19 to the membrane receptor FGFR4 was necessary for FGF19-mediated cell cycle arrest, and further proved that FGF19-mediated cell cycle arrest was via activation of p38/MAPK signaling. Through inhibitor experiments, we discovered that inhibition of FGFR4 led to down-regulation of p38 signaling even in the presence of FGF19. Meanwhile, inhibiting p38 signaling reduced the cell cycle arrest of chondrocytes induced by FGF19. Furthermore, blocking p38 signaling facilitated to retain the expression of cdk1 and cyclinb1 that had been reduced in chondrocytes by FGF19 and decreased the expression of chk1 and gadd45a that had been enhanced by FGF19 in chondrocytes. Taking together, this study is the first to demonstrate that FGF19 induces cell cycle arrest at G2 phase via FGFR4-p38/MAPK axis and enlarges our understanding about the role of FGF19 on cell cycle progression in chondrocytes.

## Introduction

Most long bones in vertebrates develop through endochondral ossification, a highly regulated process. The chondrocyte must undergo a series of processes such as proliferation, cell cycle exit, differentiation, and apoptosis to complete this process. Early studies have found that fibroblast growth factors (FGFs) trigger an intricate network of signaling that lead to chondrocyte growth inhibition and abnormal differentiation [1, 2]. FGFs inhibit chondrocyte proliferation both in vivo and in vitro [3]. FGF1/2-mediated chondrocytes cell cycle G1 arrest is an important pathway to inhibit chondrocyte proliferation [4, 5]. Dephosphorylation of p107 mediated by PP2A phosphatase and the Ras/MAPK pathway are critical events in FGF1/2-induced growth arrest [6, 7]. Not only proliferation, but FGF2 also interferes with chondrocyte differentiation. FGF2 induces significant changes in the cartilage-like phenotype of chondrocytes, such as loss of extracellular matrix and inhibition of aggrecan expression [8, 9]. However, in the face of so many members, i.e., 7 subfamilies and 22 members [10], it is still a huge challenge to find out whether and how these members affect the cell behaviors including proliferation and cell cycle.

FGF19 is an endocrine FGF with a low affinity for FGF receptors (FGFR) in comparison to other FGFs, thus requiring the co-receptor β-klotho (KLB) to stimulate cellular activity [11, 12]. FGF19 has a high selectivity for FGFR4 binding in the presence of KLB [13, 14], and studies on FGFR4 in bone development are scarce, but FGFR4 has been demonstrated to be expressed in both neonatal mouse calvaria and primary osteoblasts [15]. Since being initial discovery, FGF19 has been proposed as a candidate gene for bone diseases because of the region of the FGF19 gene on the chromosome associated with osteoporosis-pseudoglioma syndrome [14, 16]. Later, FGF19 is proved to be expressed throughout the growth plate of infants and is the major FGF ligand [17]. A recent study has shown that FGF19 can promote osteogenic differentiation and prevent bone loss caused by obesity [18]. However, research on FGF19 in cartilage development is currently at the genetic screening stage, with no in-depth examination of its unique phenotype. The mechanisms involved are still not fully understood.

The proper conduct of the cell cycle is essential for cell proliferation and differentiation and relies on regulatory mechanisms that ensure that cell cycle events occur in the correct sequence. This tightly regulated network is known as the cell-cycle control system [19, 20]. The enzyme family of cyclin-dependent kinases (cdks) is the core component of the cell-cycle control system [21]. Cdk1 interacts with cylinb1 to promote the transition from the G2 phase to mitosis [22]. Cdk1 and cylinb1 are controlled by checkpoint kinases such as checkpoint kinase 1 (chk1) and wee1, a conserved protein kinase. Cell cycle G2 arrest can be caused by cdk1 inactivation via chk1/chk2 activation [23]. By controlling the actions of cyclins and cdks, the checkpoints induce cell cycle arrest and trigger cellular responses to DNA damage at different stages [23]. Growth arrest and DNA-damage-inducible 45 alpha (gadd45a) are usually associated with growth arrest and play a role in the S phase and G2/M phase arrest. Gadd45a can block cells at the G2/M checkpoint because it can decrease cdk1 activity and prevent it from attaching to cylinb1 [24].

Some previous studies have explained how FGF1/2 can cause cell proliferation arrest by mediating cell cycle G1 arrest of chondrocytes [25]. In addition to G1/S phase arrest, FGF may also be involved in G2/M phase cell cycle arrest [26]. However, the role of FGF19 on the cell cycle of chondrocytes has not been confirmed yet. This study aims to explore the role of FGF19 in the cell cycle regulation of chondrocytes and the potential mechanisms, which might broaden our understanding of the role of FGF19 in cartilage proliferation and development.

## Results

### FGF19 decreases chondrocyte proliferation and mediates cell cycle G2 phase arrest

**Fig. 1:**
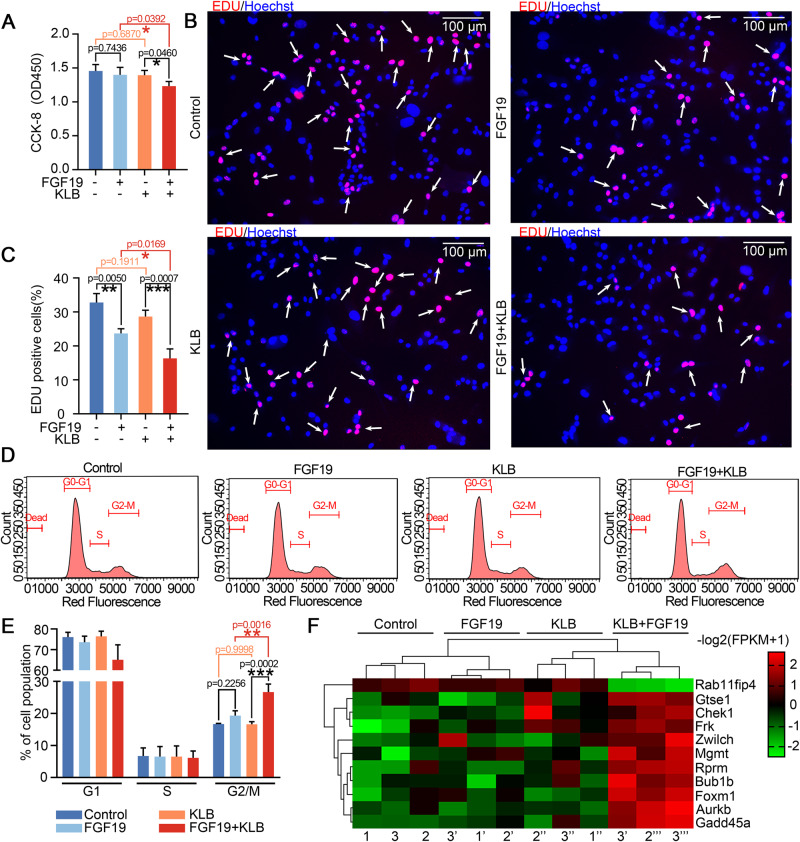
FGF19 induces cell cycle arrest of chondrocytes at the G2/M Phase. **A** CCK-8 assay showing the changes in cell viability of chondrocytes induced by 200 ng/ml FGF19 for 72 h with KLB (200 ng/ml). KLB, β Klotho. **B** EDU staining indicating DNA synthesis of chondrocytes induced by 200 ng/ml FGF19 for 72 h with KLB (200 ng/ml). **C** Quantitative analysis of EdU-positive cells in (**B**). **D** Cell cycle changes based on flow cytometry showing cell cycle G2/M phase arrest of chondrocytes induced by 200 ng/ml FGF19 for 72 h in the presence of KLB (200 ng/ml). **E** Quantitative analysis of cell populations in (**D**). **F** Pheatmap showing the related gene changes of cell cycle arrest in chondrocytes based on RNA sequencing. Rows represent genes, and columns represent different groups. The red indicates the upregulation of gene expression, while the green indicates the downregulation of genes. The datasets were presented in FPKM and were clustered using the online R package. Three independent cell isolates yielded three pairs of lysate samples (*n* = 3). Each pair sprang from identical maternal cells. The data in **A**, **C** and **E** were presented as the means ± SD, and the significant data presented in **A**, **C** and **E** were based on two-tailed Student’s *t*-tests. Experiments presented in **A**, **B**, **D** and **F** were based on at least three independent repeats (*n* ≥ 3).

To find out the role of FGF19 on the cell cycle of chondrocytes, the effect of FGF19 on chondrocyte cell viability was first investigated in the presence/absence of KLB, an important accessory protein that assists in the binding of FGF19 to its receptor [11, 12]. The result showed that, without KLB, FGF19 did not induce the change of cell viability of chondrocytes, but with the help of KLB, FGF19 significantly reduced the cell viability by using CCK8 assay (Fig. 1A). We then performed EDU staining and found that FGF19 could reduce DNA synthesis and thus impair proliferation of the chondrocytes, whether with or without KLB (Fig. 1B and C), especially, with the help of KLB, the role of FGF19 in decreasing DNA synthesis appeared stronger. We then used flow cytometry to investigate how FGF19 altered the cell cycle in chondrocytes. (Fig. 1D), and we found that significant changes in the G2 phase appeared in chondrocytes induced by FGF19. This result confirmed that chondrocytes at ~28% were arrested at the G2 phase in the FGF19 group with the help of KLB relative to 16.70% in the KLB control group (Fig. 1E). By using RNA sequencing, we screened out all changed gene candidates in the cell cycle arrest (cell cycle checkpoint) and clustered these genes by pheatmap (Fig. 1F). Taking together, these results suggested that FGF19 triggered chondrocyte cell cycle G2 phase arrest.

### FGF19 mediates cell cycle arrest by down-regulation of cdk1/cylinb1 and up-regulation of chk1 and gadd45a

**Fig. 2:**
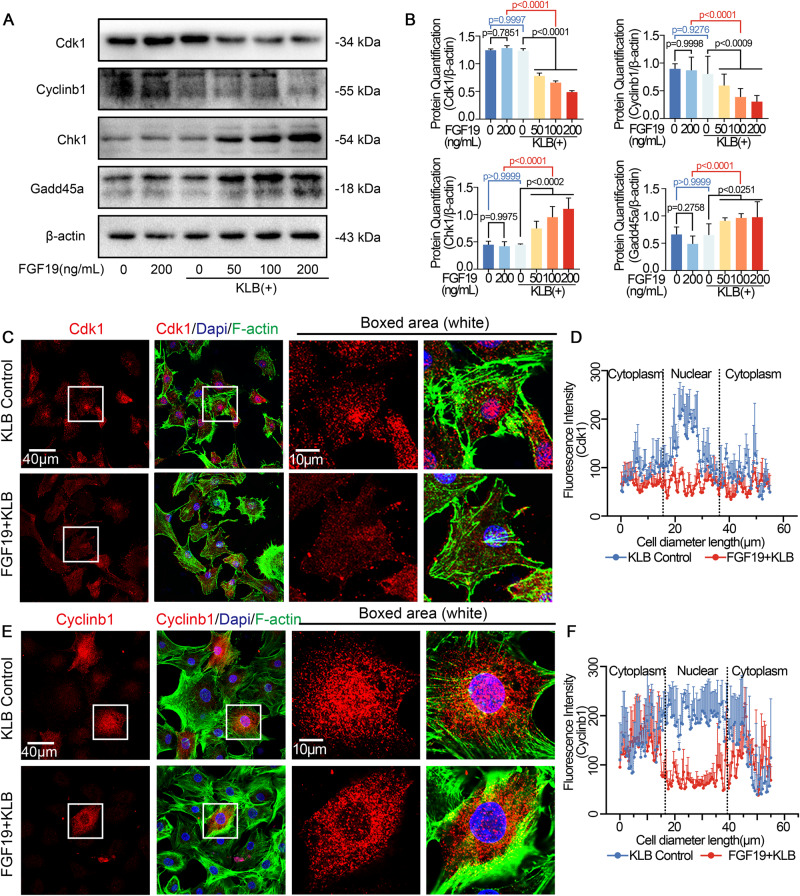
FGF19 mediates cell cycle arrest of chondrocytes at the G2 phase by down-regulation of cdk1 and cylinb1 and up-regulation of chk1 and gadd45a. **A** Western blot showing the expression change of cdk1, cylinb1, chk1 and gadd45a in chondrocytes stimulated with 0, 50, 100 and 200 ng/ml FGF19 for 72 h in the presence of the corresponding concentration of KLB (0, 50, 100 and 200 ng/ml). **B** Quantitative analysis presenting the protein expression of cdk1, cylinb1, chk1 and gadd45a in chondrocytes in (**A**). **C** Representative immunofluorescent images showing the reduced expression of cdk1 in chondrocytes induced by 200 ng/ml FGF19 for 72 h with KLB (200 ng/ml). Cytoskeleton, green; Cdk1, red; nucleus, blue. **D** Linear fluorescent quantification presenting the distribution of cdk1 in chondrocytes in (**C**). **E** Representative immunofluorescent images showing the reduced nuclear expression of cylinb1 in chondrocytes induced by 200 ng/ml FGF19 for 72 h with KLB (200 ng/ml) by CLSM. Cytoskeleton, green; Cylinb1, red; nucleus, blue. **F** Quantification of the linear fluorescent protein of cylinb1 showing the cylinb1 intracellular distribution in chondrocytes in (**E**). The data in **B**, **D** and **F** are presented as the means ± SD. The significant analysis in **B** was based on two-tailed Student’s *t*-tests. All results in **A**, **C** and **E** were obtained from three independent experiments (*n* = 3).

Cdk1 and cyclinb1 are recognized to be the driving force for cells to enter mitosis [27]. The cell cycle would stop at G2 phase if cdk1 and cyclinb1 activity were inhibited. Therefore, we first detect the expression of cdk1 and cyclinb1 induced by FGF19 and KLB in chondrocytes. The result showed that FGF19 at different concentrations reduced the protein expression of cdk1 and cyclinb1 (Fig. 2A and B). Meanwhile, from our RNA sequencing results in Fig. 1F we found that the transcription level of cell cycle checkpoint kinases, chk1 or Chek1, and DNA damage response protein, gadd45a, was increased. Therefore, we also verified the protein expression of chk1 and gadd45a (Fig. 2A and B). The results demonstrated that chk1 and gadd45a protein expression was enhanced in chondrocytes stimulated by FGF19 with KLB. In order to enter the mitotic stage, cdk1 and cyclinb1 must translocate into the nucleus to initiate mitosis [20]. We performed immunofluorescence staining to explore whether FGF19 could regulate the nuclear accumulation of cdk1 and cyclinb1. As expected, the nuclear expression of cdk1 and cyclinb1 in chondrocytes induced by FGF19 was significantly decreased (Fig. 2C and E). Most cyclins exhibit drastic concentration changes during the cell cycle to help generate oscillatory changes in cdk activity. Cyclinb1 expression begins to rise near the G2 phase and peaks at mid-mitosis. Therefore, high expression of cyclinb1 can only be observed in cells approaching the G2 phase [28]. In addition, fluorescence intensity quantification showed that in the presence of KLB, less cdk1 and cyclinb1 protein accumulated in the nucleus of chondrocytes stimulated by FGF19. (Fig. 2D and F). These results indicated that FGF19 down-regulated the expression of cdk1 and cyclinb1 and decreased their entry into the nuclei; meanwhile, FGF19 up-regulated the expression of chk1 and gadd45a, thus forcing more chondrocytes to stay at G2 phase.

### FGF19 mediates cell cycle arrest at G2 phase in chondrocytes via FGFR4

**Fig. 3:**
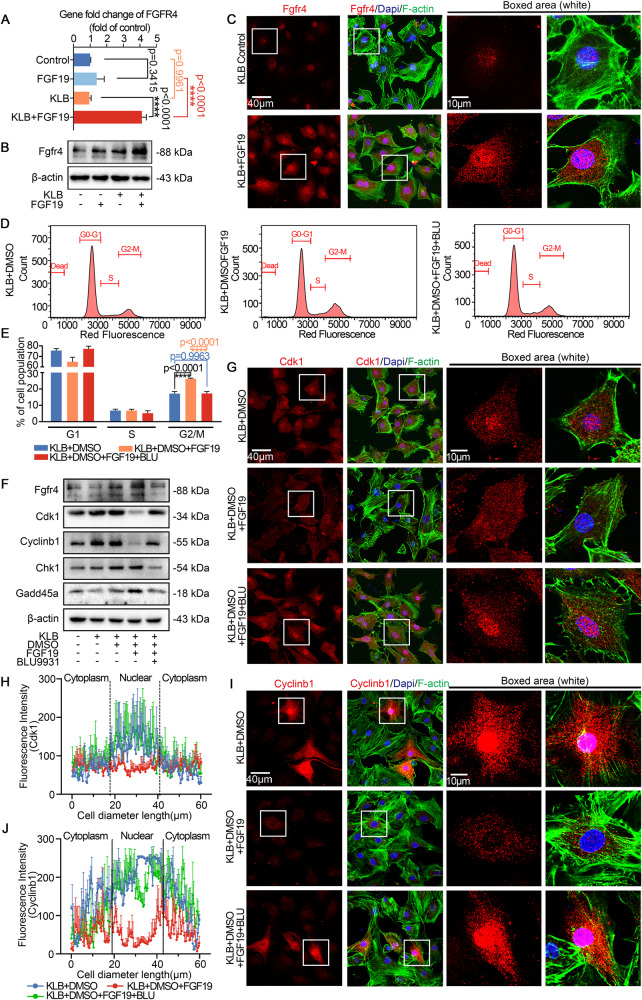
FGF19 induces cell cycle arrest at G2 phase through FGFR4. **A** Representative q-PCR results showed that FGF19 (200 ng/ml) and KLB stimulate chondrocytes could increase mRNA expression of FGFR4. **B** Representative western blots confirming the change in protein expression of FGFR4 in chondrocytes induced by 200 ng/ml FGF19 with KLB. **C** Representative immunofluorescent pictures presenting chondrocytes treated with 200 ng/ml FGF19 and KLB with higher FGFR4 expression. Cytoskeleton, green; FGFR4, red; nucleus, blue. **D** Flow cytometry analysis demonstrated that 5 μM BLU9931 attenuated 200 ng/ml FGF19-induced cell cycle G2 phase arrest in the presence of KLB. **E** Quantitative analysis of cell populations in (**D**). **F** Representative western blots showed that FGF19 (200 ng/ml) combined with KLB stimulation of chondrocytes causes changes in the expression of FGFR4, cdk1, cylinb1, chk1 and gadd45a in the presence or absence of BLU9931 (5 μM). **G** Representative immunofluorescent pictures presenting the increase of cdk1 in chondrocytes after pretreatment with 5 μM BLU9931 for 2 h in the presence of FGF19 (200 ng/ml) and KLB for 72 h. Cytoskeleton, green; Cdk1, red; nucleus, blue. **H** Quantification of the linear fluorescent intensity of cdk1 in chondrocytes shown in (**G**). **I** Representative immunofluorescent pictures presenting chondrocytes with higher cylinb1 expression pretreated with 5 μM BLU9931 for 2 h in the presence of FGF19 and KLB for 72 h. Cytoskeleton, green; Cylinb1, red; nucleus, blue. **J** Quantification of the linear fluorescent intensity of cylinb1 in chondrocytes shown in (**I**). The data in **A**, **E**, **H,** and **J** were presented as the means ± SD, and the significant data presented in **A** and **E** were based on two-tailed Student’s *t*-tests. Experiments presented in **B**–**D**, **F**, **G** and **I** were based on at least three independent repeats (*n* ≥ 3).

The ability of FGF19 to bind to FGFR1, FGFR2, FGFR3, and FGFR4 is well known, but FGFR4 is its preferred receptor [13, 14]. We first performed qPCR to identify changes in FGFRs in chondrocytes after treatment with FGF19 in the presence of KLB (Figs. S1 and 3A). The qPCR results showed that treating chondrocytes with 200 ng/ml FGF19 greatly increased the expression of FGFR4 (Fig. 3A). Although we discovered that FGF19 might increase FGFR1 gene expression (Figure S1), but it has been reported that FGF19 mainly binds to FGFR4 to activate the cellular response [11]. At the protein level, we used a western blot assay to assess FGFR4 protein alterations in chondrocytes stimulated by FGF19 for 72 h. The results indicated that FGFR4 was significantly increased (Fig. 3B). Quantification further confirmed this result (Fig. S2). The distribution of FGFR4 in chondrocytes was examined and we found that the expression of FGFR4 was increased in the KLB + FGF19 group by immunofluorescence (Fig. 3C). To further investigate whether FGF19-mediated cell cycle arrest of chondrocytes was via FGFR4, we used BLU9931, a specific inhibitor of FGFR4 [29]. By using flow cytometry, we found that BLU9931 could disrupt FGF19-mediated G2 phase arrest (Fig. 3D and E). Quantification of flow cytometry indicated that with the help of KLB, chondrocytes at ~26.20% were arrested at G2 phase induced by FGF19, while the participation of BLU9931 reduced the number of chondrocytes at G2 phase to ~16% (Fig. 3E), indicating the significant inhibitory role of BLU9931 in preventing the binding between FGF19 and FGFR4, thereby weakening the entry of FGF19 into cells. To confirm whether the binding inhibition influences the cell cycle regulatory proteins, i.e., cdk1, cyclinb1, chk1 and gadd45a, we performed further experiments. Western blot analysis showed that BLU9931 pretreatment could reverse the down-regulation of cdk1 and cyclinb1 and greatly impair the up-regulation of chk1 and gadd45a induced by FGF19 (Fig. 3F). These results were further supported by the quantification. (Fig. S3). We next performed immunofluorescent staining about cdk1 and cyclinb1, and the results revealed that, after pretreatment with BLU9931, the role of FGF19 on chondrocytes was largely blocked and the normal nuclear accumulation of cdk1 and cyclinb1 was recovered (Fig. 3G and I). Linear quantification of cdk1 and cyclinb1 confirmed these results (Fig. 3H and J). Taking together, these results demonstrated that FGF19-mediated cell cycle G2 phase arrest in chondrocytes through FGFR4.

### FGF19 mediates cell cycle G2 phase arrest in chondrocytes by activating p38 signaling

**Fig. 4:**
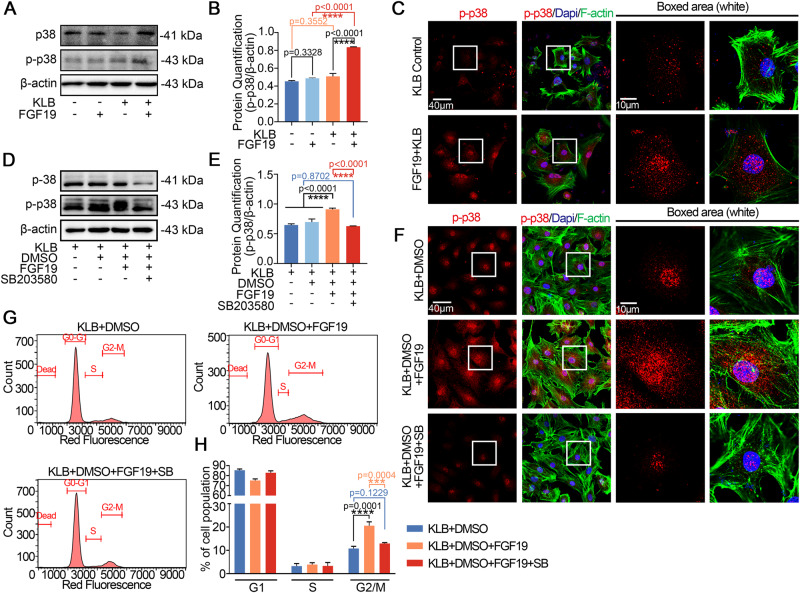
FGF19 mediates cell cycle arrest at G2 phase by activating p-p38 signaling. **A** Western blots showing the activated p-p38 protein expression in chondrocytes treated with 200 ng/ml FGF19 for 6 h in the presence of KLB (200 ng/ml). **B** Quantification analysis of p-p38 protein expression in (**A**). **C** Representative immunofluorescence images showed that 200 ng/ml FGF19 with KLB-induced intranuclear accumulation of p-p38. **D** Representative western blots showed that SB203580 reduced p-p38 expression induced by 200 ng/ml FGF19 and 200 ng/ml KLB. **E** Histogram showing the quantitative analysis of p-p38 protein expression in (**D**). **F** Representative IF images showing protein changes of p-p38 in chondrocytes after pretreatment with 10 μM SB203580 for 2 h in the presence of FGF19 (200 ng/ml) and KLB. Cytoskeleton, green; p-p38, red; Nucleus, blue. **G** Flow cytometry analysis showing that 10 μM SB203580 attenuated 200 ng/ml FGF19-mediated cell cycle G2 phase arrest in the presence of KLB. **H** Quantitative analysis of cell populations in (**G**). The data in **B**, **E** and **H** are presented as the means ± SD and significant data presented were based on two-tailed Student’s *t*-tests. Experiments presented in **A**, **C**, **D**, **F** and **G** were based on at least three independent repeats (*n* ≥ 3).

We used western blot to detect the potential signaling pathways that may have changed in order to explore the mechanisms of FGF19-mediated chondrocytes cell cycle arrest. The results showed that p-p38 was up-regulated after being treated with 200 ng/ml FGF19 with KLB in chondrocytes for 6 h (Fig. 4A), and quantification confirmed the result (Fig. 4B). In addition, FGF19 involvement did not significantly alter other signaling pathways including ERK/p-ERK (Fig. S4) and AKT/p-AKT (Fig. S5). By using confocal laser microscopy (CLSM), we showed that enhanced p-p38 protein accumulated in the nuclei of chondrocytes after treatment with FGF19 and KLB (Fig. 4C). The above results guided us to further investigate the importance of p-p38 signaling in FGF19-mediated cell cycle arrest. SB203580, the p-p38 signaling-specific inhibitor [30], was used to confirm the function of p-p38 signaling in FGF19-mediated cell cycle changes. From western blot, we can see that SB203580 significantly downregulated the expression of total and phosphorylated p38 signaling induced by FGF19 (Fig. 4D). Quantification further confirmed these results (Fig. 4E). From the immunofluorescence, we then revealed that SB203580 reduced the expressions of p-p38 signaling in chondrocytes with FGF19 (Fig. 4F). By using flow cytometry, SB203580 was found to drastically reduce the cell cycle arrest in chondrocyte population (Fig. 4G & H). The proportion of chondrocytes arrested in the G2 phase decreased from 25% in the KLB + FGF19 group to 12% in the KLB + FGF19 + SB203580 group (Fig. 4H). All together, these results indicated the importance of p-p38 signaling on FGF19-induced cell cycle G2 phase arrest.

### FGF19 mediates cell cycle G2 phase arrest in chondrocytes via FGFR4-p38/MAPK axis

**Fig. 5:**
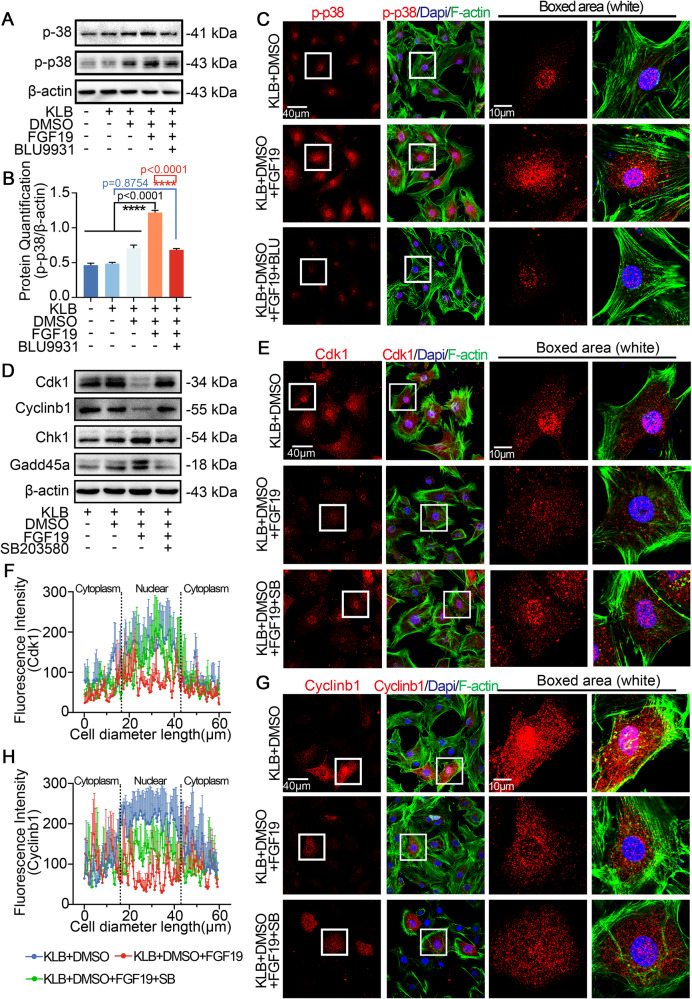
FGF19 mediates cell cycle arrest at G2 phase by activating FGFR4-p38/MAPK axis. **A** Representative western blots showed that FGF19 (200 ng/ml) combined with KLB stimulation of chondrocytes causes changes in the expression of p-p38 for 6 h in the presence or absence of BLU9931 (5 μM). **B** Quantification analysis to confirm the protein changes in (**A**). **C** Representative IF images presenting chondrocytes pretreated with 5 μM BLU9931 for 2 h and stimulated with 200 ng/ml FGF19 and KLB for 6 h showed less nuclear translocation of p-p38 in comparison to the group treated solely with FGF19 and KLB. (Cytoskeleton, green; p-p38, red; Nucleus, blue). **D** Representative western blots showed that FGF19 (200 ng/ml) combined with KLB stimulation of chondrocytes causes changes in the expression of cdk1, cylinb1, chk1 and gadd45a in the presence or absence of SB203580 (10 μM). **E** Representative IF pictures presenting chondrocytes with higher cdk1 expression pretreated with 10 μM SB203580 for 2 h in the presence of 200 ng/ml FGF19 and KLB. Cytoskeleton, green; Cdk1, red; Nucleus, blue. **F** Quantification of the linear fluorescent intensity of cdk1 in chondrocytes shown in (**E**). **G** Representative IF pictures presenting chondrocytes with higher cylinb1 expression after pretreatment with 10 μM SB203580 for 2 h in the presence of 200 ng/ml FGF19 and KLB. Cytoskeleton, green; Cylinb1, red; Nucleus, blue. **H** Quantification of the linear fluorescent intensity of cylinb1 in chondrocytes shown in (**G**). The data in **B**, **F** and **H** are presented as the means ± SD, and the significant data presented in **B** were based on two-tailed Student’s *t*-tests. Experiments presented in **A**, **C**–**E** and **G** were based on at least three independent repeats (*n* ≥ 3).

To investigate the relationship between FGFR4 and p38/MAPK signaling in FGF19-mediated cell cycle arrest, we first applied BLU9931 and found that BLU9931 treatment could decrease the expression of p-p38 signaling by western blot (Fig. 5A and B). BLU9931 could also diminish the p-p38 nuclear accumulation stimulated by FGF19, according to immunofluorescence (Fig. 5C). These findings demonstrated a connection between FGF19-induced FGFR4 and p38 signaling in chondrocytes.

To substantiate the role of p-p38 signaling in cell cycle arrest, we applied SB203580 to analyze the protein change of cdk1, cyclinb1, chk1 and gadd45a. By western blot, SB203580 retained the expression of cdk1 and cyclinb1 reduced by FGF19 in chondrocytes and impaired the expression of chk1 and gadd45a enhanced by FGF19 in chondrocytes (Fig. 5D). The quantification further confirmed these results (Fig. S6). Considering the important role of cdk1 and cyclinb1 in the cell cycle of chondrocytes, we performed immunofluorescence (Fig. 5E and G), and the results demonstrated that SB203580 could maintain cdk1 and cyclinb1 expression that had been decreased by FGF19. From linear fluorescence quantification, we can see that the nuclear expressions of cdk1 and cyclinb1 were enhanced in the BLU9931 + FGF19 group (Fig. 4F and H). Taking together, it was shown that the FGFR4-p38/MAPK axis is crucial for controlling the FGF19-induced cell cycle G2 phase arrest in chondrocytes.

## Discussion

As a hormone-like secreted factor, FGF19 is crucial for growth and energy metabolism [11]. Recently, studies on FGF19 in the skeletal system, such as skeletal muscle and osteogenic differentiation, have been published for the first time. Benoit B et al. reported that FGF19 increased myodynamia in mice by enhancing skeletal muscle weight. Furthermore, human myotube size was considerably increased in vitro by FGF19 via phosphorylating ERK1/2 [31]. Guo A. et al. showed that FGF19 promotes osteogenic differentiation by regulating the Wnt/β-catenin pathway to attenuate obesity and ageing-induced bone loss [18, 32]. Additionally, our team has just reviewed FGF19’s function and therapeutic potential in skeletal development and diseases, describing its potential physiological role in cartilage and endochondral ossification, and its role in bone diseases including Apert syndrome, osteoarthritis, and osteoporosis [10]. In the study, we concentrated on how FGF19 affects the cell cycle and proliferation of chondrocytes. Our results show that FGF19 activates the MAPK P38 pathway via FGFR4, inhibiting cdk1/cyclinb1 protein and ultimately leading to cell cycle G2 phase arrest. This study provides evidence that FGF19 can be a new direction for cartilage-related research.

Chondrocyte proliferation and differentiation rely on cell cycle regulation. Many studies have shown that the transition of chondrocytes from proliferation to hypertrophic differentiation in vivo necessitates cell cycle arrest and exit [33–36]. Although FGFs induce cell proliferation in most cell types, they inhibit chondrocyte proliferation [37]. These studies have focused on proliferation inhibition caused by the blockade of the G1 or G2 phase induced by FGF1/2 treatment of primary chondrocytes or chondrocyte lines [26]. Proteins, p107 and p130, are required for FGF inhibition of chondrocyte proliferation. Early p107 dephosphorylation is a critical event in FGF-induced growth arrest, which is mediated by PP2A phosphatase. Unlike p107, p130 dephosphorylation occurs late, to sustain FGF-induced proliferation inhibition [4, 6, 26]. In our experiments, we found for the first time that FGF19 mediates G2 phase arrest in primary chondrocytes. This result is consistent with the negative effect of the FGF family on chondrocyte growth.

Previous studies have demonstrated that downregulation of cdk1/cylinb1 activity in FGF1-induced G2 arrest of the chondrocyte cycle is a direct consequence of FGF signaling [26]. The activation of cdk1 is a critical event for cell mitotic entry. Although cdk1 levels remain constant throughout the cell cycle, it is only active during mitosis because it is regulated by several mechanisms, including cyclin binding and phosphorylation [38]. Cylinb1 is cdk1’s primary partner, and its concentration rises as cells approach mitosis, peaking in metaphase [39]. FGF19 stimulation not only decreased the expression of cylinb1 and cdk1 but also blocked their nuclear import. Cellular G2 arrest and downregulation of cylinb1/cdk1 activity are known features of cellular DNA damage responses [40]. DNA damage stress triggers the G2 checkpoint, which is activated by ATM/ATR and chk1/2 kinases and results in cdk1 inhibition [41, 42]. When gadd45a is activated, it binds cdk1 more tightly and pushes cdk1 away from the cdk1/cylinb1 complex [43]. Free cylinb1 undergoes proteasome-mediated degradation, resulting in G2/M cell cycle arrest [44]. Our study found that the expression of chk1 and gadd45a increased following FGF19 stimulation, which may be related to the chondrocyte DNA damage brought on by FGF19 stimulation.

FGFRs are transmembrane receptors with extracellular, transmembrane, and intracellular domains [11]. FGF19 requires the presence of KLB to bind and activate FGFR4 efficiently [45, 46]. Many cellular processes are influenced by FGF19/FGFR4 signaling, including cell proliferation, migration, and differentiation [47]. In the study, we investigated the FGFRs’ expression in chondrocytes treated with FGF19 and demonstrated that FGFR4 expression rose dramatically as a result. BLU9931 is an irreversible and highly selective small molecule inhibitor of FGFR4 [48]. Our experiments found that after BLU9931 blocked FGFR4, the G2-phase arrest effect of FGF19 on chondrocytes was abolished, and the activation of p-p38 by FGF19 was also inhibited. As a result, FGFR4 is one of the critical pathways by which FGF19 activates the MAPK p38 signaling pathway to cause cycle arrest. However, FGF19 can signal via KLB binding to FGFR1-3 at supraphysiological concentrations, more research is needed to determine whether FGF19 affects the cell cycle of chondrocytes via other receptors.

To further understand the mechanism by which FGF19 stimulates chondrocyte cycle arrest, we measured the signaling pathways, which are associated with FGFs, like MAPK p38, MAPK ERK1/2 and AKT signaling pathway. The results revealed that FGF19-induced MAPK p38 signaling in chondrocytes. The MAPK p38 signaling cascade, once activated, influences the cell cycle by affecting the timing of cell cycle entrance and checkpoint termination [49, 50]. In response to cellular stress, p38 frequently acts as a “brake”, inhibiting cell cycle transitions. MAPK p38 signaling activation can result in the degradation of CDC25B and CDC25C phosphatases, both required for cdk1 activation [51, 52]. As a result, cdk1/cylinb1 activation is blocked, thereby preventing G2 progression. p38 is also associated with gadd45a protein [53]. P38 can directly phosphorylate p53, thereby upregulating downstream effectors like gadd45a, which would contribute to p38 activation. A positive feedback loop is formed to induce cell cycle arrest [22]. Previous studies have also shown that chk1 can synergize with p38 to induce cell cycle checkpoint arrest [54]. In our study, FGF19 activated MAPK p38, and with specific blocker SB203580, cell cycle arrest was rescued, cdk1 and cylinb1 expression were also picked up, and FGF19 activation of chk1 and gadd45a was eliminated. All of the findings point to FGF19 inducing G2 arrest in primary chondrocytes via MAPK p38 activation (Fig. 5).

**Fig. 6:**
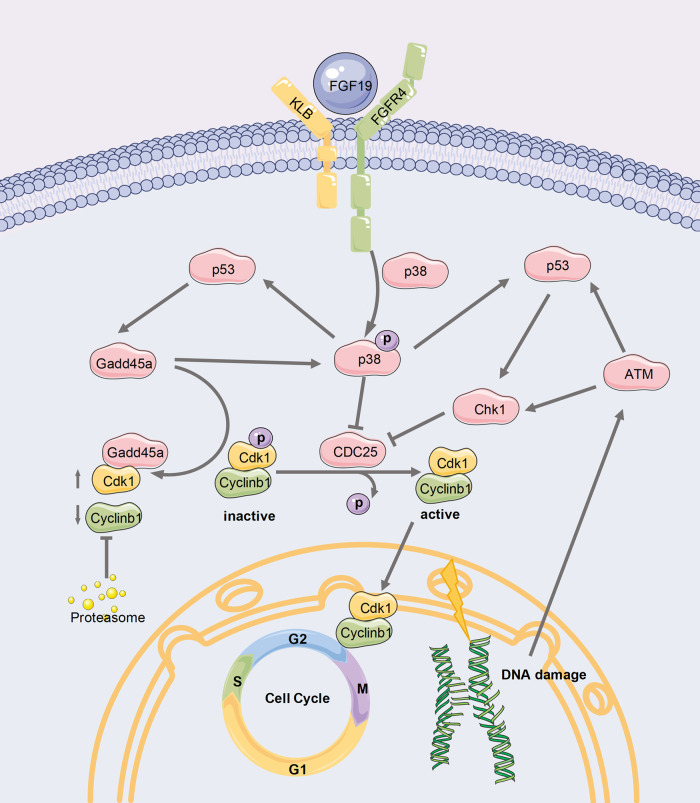
Schematic diagram showing the mechanism of FGF19-induced G2 phase arrest in primary chondrocytes. FGF19 activates p-p38 signaling through FGFR4, increases chk1 and gadd45a activity, inhibits cdk1 and cylinb1 expression and nuclear accumulation, and consequently induces cell cycle arrest of chondrocytes at the G2/M Phase.

In conclusion, we demonstrate for the first time that FGF19 induces G2 arrest of the chondrocyte cell cycle, revealing the mechanism by which FGF19 activates FGFR4-p38/MAPK axis, leading to inhibition of cylinb1/cdk1 activity (Fig. 6). These findings deepen our comprehension of FGF19’s function in articular cartilage. FGF19 may be a therapeutic target for cartilage diseases.

## Materials and methods

### Cell culture

The Sichuan University Institutional Review Board (IRB) authorized all protocols and the materials utilized in this investigation were acquired in accordance with ethical standards (IRB at the West China Hospital of Stomatology, No. WCHSIRB-OT-2020-048). Primary chondrocytes were isolated from newborn C57/BL mice articular cartilage as previously described [55]. Briefly, after dissociating articular cartilage, the surrounding connective tissue was stripped and the obtained articular cartilage was rinsed with 1 × phosphate-buffered saline (PBS, SH30256, HyClone, UT, USA) containing 1% penicillin–streptomycin solution (PS, SV30010, HyClone, UT, USA). Trypsinize the harvested tissue in 0.25% protease solution for 30 min. After cleaning with PBS, add 0.2% type II collagenase for overnight incubation. The next day, the digested tissue suspension was neutralized by an equal amount of fresh 10% fetal bovine serum DMEM (SH30070, HyClone, UT, USA) placed in a centrifuge tube and centrifuged at 150×*g* for 5 min. The cultured chondrocytes were suspended in the culture medium and plated in Petri dishes.

### Cell counting Kit-8 assay

In 96-well plates, chondrocytes (passages 1–2) were implanted at a density of 2000 per well. The cell counting kit-8 (CK04, Dojindo, Kumamoto, Japan) was used to measure the cell proliferation rates after treatment with FGF19 for 72 h (#100-32, Pepro-tech, NJ, USA) at the concentrations of 200 ng/ml in the presence of the same concentration of KLB (2619-KB, Bio-Techne, Shanghai, China). The CCK-8 working solution (10 μl) was introduced to each well after replacing 100 μl of the FGF19-free culture medium. A multi-mode microplate reader was used to measure the optical density at 450 nm after incubating the plates at 37 °C for 2 h.

### 5-Ethynyl-2′-deoxyuridine (EdU) assay

The study used the EdU kit (KGA335-100, EDU-647, KeyGen, Nanjing, China) to perform the EdU assay. Firstly, EdU working solution (1:1000) was added to the chondrocytes stimulated by FGF19 and cultured at 37 °C for 2 h. Next, the 4% paraformaldehyde was used to fix the cells for 30 min and then the cells were treated with 0.5% Triton X-100. According to the manufacturer’s instructions, click reaction solution was added to the cell for 30 min in a dark environment before being exposed to Hoechst solution. We captured images with a fluorescent microscope (Olympus, Japan) and ImageJ software (NIH, Bethesda, MD, USA) was used to cell counting.

### Flow cytometry analysis

We used flow cytometry to analyze the cell cycle changes. Cells were seeded into six-well plates at a density of 5 × 10^5^ cells per well. The next day, the cells were treated with or without 200 ng/ml FGF19 and KLB. The cells were collected after 24 h of treatment and then fixed with 70% ethanol. The fixed cells were stored at 4 °C overnight. The cells were rinsed with cold PBS after centrifugation (600×*g*, 5 min, 4 °C). Subsequently, 500 μl PI/RNase (KGA511, KeyGen, Nanjing, China), a dye working solution prepared in advance, was added to the cells to stain the DNA, and the cells were incubated for 30 min in the dark. Flow cytometry (GuavaeasyCyte, Merck, MA, USA) was used to analyze the stained cells, and Guava InCyte software was used to examine the percentage of cells present in the different cell cycle phases.

### RNA sequencing

After chondrocytes were treated with FGF19 and KLB for 72 h, their cell lysates were harvested using Trizol reagent (15596018, Thermo, MA, USA). RNA Nano 6000 Assay Kit with a Bioanalyzer 2100 system was used to evaluate the RNA integrity. They were then sent to Shanghai Lifegenes Biotechnology Co., Ltd. (Shanghai, China) for transcriptome analysis. For RNA-seq analysis, GO enrichment analysis of differentially expressed genes was performed using the DAVID database and implemented using the KEGG database (http://www.genome.jp/kegg/pathway.html). The procedures were described in depth in our previous publication [56, 57]. The significance levels were set to be 0.05 and |FoldChange | >1.5.

### Western blot analysis

Chondrocytes were treated with FGF19 and KLB at 50, 100, and 200 ng/ml for 72 h, respectively. BLU9931 (5 μM, 5387760001, Sigma, MO, USA) and SB203580 (10 μM, A8254, APExBIO, TX, USA) was added 2 h prior to FGF19 and KLB treatment as previously described [58, 59]. RIPA lysis buffer (68117726, Biosharp, Hefei, China) was used to extract protein. Then we used a BCA protein assay kit to create standard curves and measure protein concentration. The sample volume contained 20 ng protein and was separated by the 10% SDS–PAGE gel. By using sandwich method, separated protein on the gel was transferred to the PVDF membrane. After blockage for 2 h with 5% skim milk, the PVDF membranes were incubated with antibodies rabbit anti-Cdk1 (1:1 000,19532-1-AP, Proteintech, Wuhan, China), rabbit anti-Cylinb1 (1:800, #4138, CST, MA, USA), rabbit anti-Chk1 (1:800,380200, Zenbio, Chengdu, China), rabbit anti-Gadd45a (1:800, 511768, Zenbio, Chengdu, China), rabbit anti-FGFR4 (1:800, 381880, Zenbio, Chengdu, China), rabbit anti-p38 (1:800, 9212, CST, MA, USA), rabbit anti-p-p38(Thr180/Tyr182) (1:800, 9211, CST, MA, USA) and Mouse anti-β-actin (1:1 000, sc-47778, Santa Cruz, USA) overnight at 4 °C. Place the PVDF membrane in the anti-IgG-HRP and incubate for 2 h after rinsing it with TBST (0.1% Tween-20/TBS) for 4 times. Super signal reagent (32106, Pierce, IL, USA) was used to obtain the blots’ signals. Quantitative analysis was performed with Image-J software.

### Immunofluorescence staining

Cells were seeded and cultured for 12 h in the dish available for CLSM as previously described [60, 61]. After treatment with FGF19 and KLB for 72 h, the cells were fixed with 4% cold paraformaldehyde. Next, the cells were washed with PBS and permeabilized with 0.5% Triton X-100 for 15 min. 5% bovine serum albumin was used to block the cells for 1 h. Then the cells were incubated with antibodies Cdk1 (1:200, 19532-1-AP, Proteintech, Wuhan, China), Cylinb1 (1:200, #4138, CST, MA, USA), FGFR4 (1:200, 381880, Zenbio, Chengdu, China) and p-p38 (Thr180/Tyr182) (1:200, 9211, CST, MA, USA) overnight at 4 °C. The dishes were incubated with Alexa Fluor 647-conjugated anti-rabbit fluorescent secondary antibody (1:200, ab150075, Cambridge, UK) on the next day for 2 h at RT. Phalloidin (FITC, 1:40, A12379, Thermo, USA) and Dapi (1:100, D9642, Sigma, MO, USA) was used to stain the cytoskeleton and the nuclei. CLSM (Olympus, FV3000, Japan) was used to obtain images of the stained cells.

### Quantitative real-time polymerase chain reaction (qRT-PCR)

Total RNA was isolated from cells after 72 h of treatment with FGF19 and KLB using the RNeasy Plus Mini Kit (74136, Qiagen, CA, USA). The Nanodrop spectrophotometer (Thermo Fisher Scientific, MA, USA) was used to quantify the extracted RNA samples. Using the cDNA synthesis kit (K1621-RevertAid, Mbi, MD, USA), total RNA was reverse-transcribed into cDNA. The iCycler (Bio-Rad, Hercules, CA, USA) was used to perform quantitative RT-PCR reactions according to the operating method. The expression of FGFRs genes was calculated using GAPDH as the internal control by a ΔΔCt method.

### Statistical analysis

For statistical analysis, Graphpad Prism 8.0 and SPSS 22.0 were utilized. Based on the results of at least three individual experiments (*n* ≥ 3), the experimental data were given as mean ± standard deviation (SD). The data statistical significance was analyzed by independent Student’s *t*-test. The critical significance level was set to 0.05.

## Supplementary information

Supplementary figures

Original data files

## Data Availability

Any data generated in this study are available from the corresponding author upon request in addition to source data.
