# Supplementary material for: FGF19 induces the cell cycle arrest at G2-phase in chondrocytes
Source: Cell Death Discov. 2023 Jul 15;9:250. doi: 10.1038/s41420-023-01543-6 (PMC10349815; doi:10.1038/s41420-023-01543-6)
Supplement: Supplementary file 1 — Supplementary figures [file 41420_2023_1543_MOESM1_ESM.doc]

Supplementary figures for

**FGF19 induces the cell cycle arrest at G2-phase in chondrocytes**

Hao Chen, Jiazhou Li, Caixia Pi, Daimoguo, Demao Zhang, Xuedong Zhou, Jing Xie

**1. Supplementary figures**

**Figure S1**

**
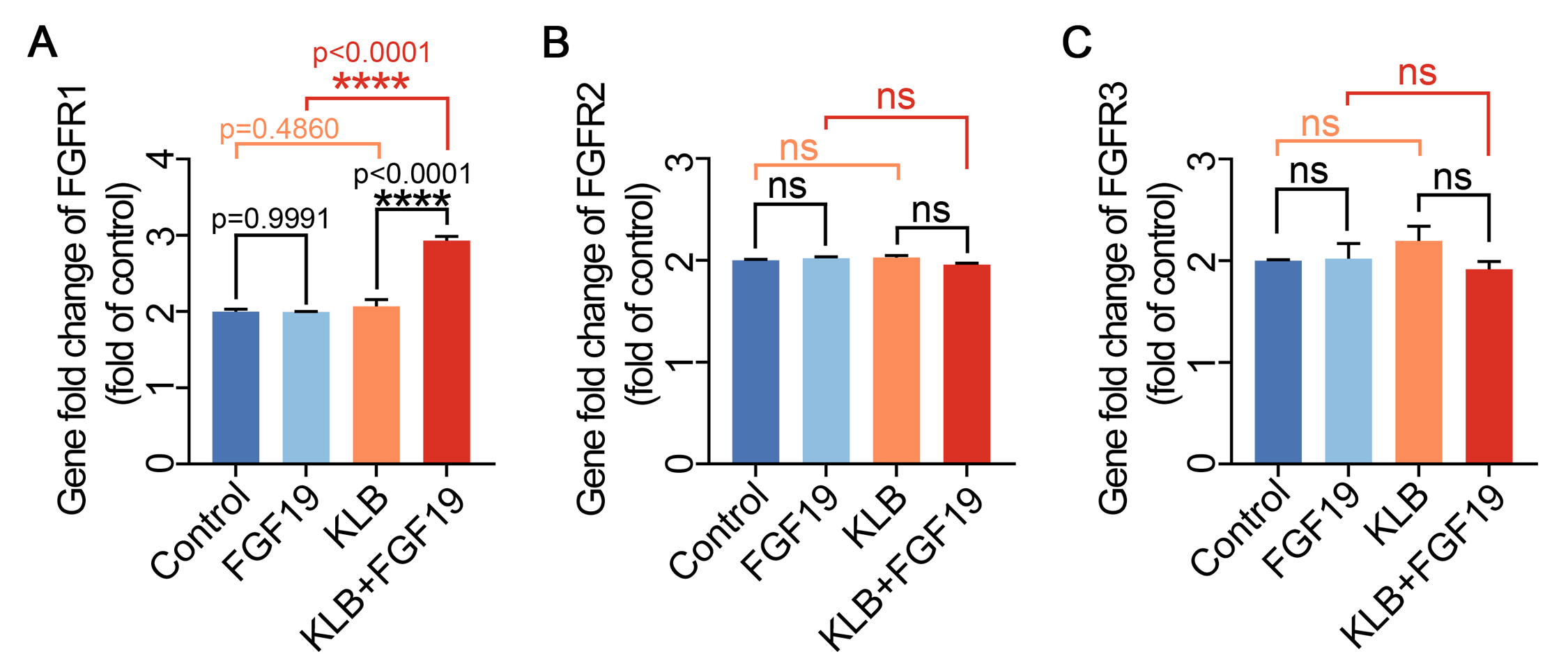
**

**Figure S1. Effect of FGF19 on FGFRs in chondrocytes.** q-PCR results showing the change of mRNA expressions of FGFRs in chondrocytes inuduced by FGF19 (200 ng/ml) in the presence of KLB (200 ng/ml) for 72 h. The results were based on three independent experiments (n = 3). All significance data presented were based on two-tailed Student’s t tests.

**Figure S2**


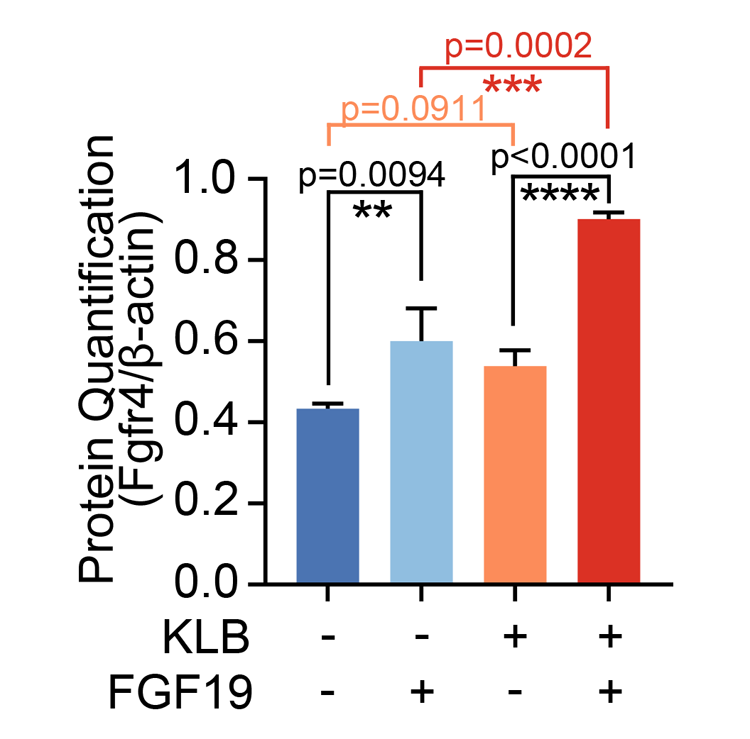


**Figure S2.** The graph showing the quantification of FGFR4 protein expression in chondrocytes in **Figure 3(B)**. The results were based on three independent experiments (n = 3). All significance data presented were based on two-tailed Student’s t tests.

**Figure S3**

**
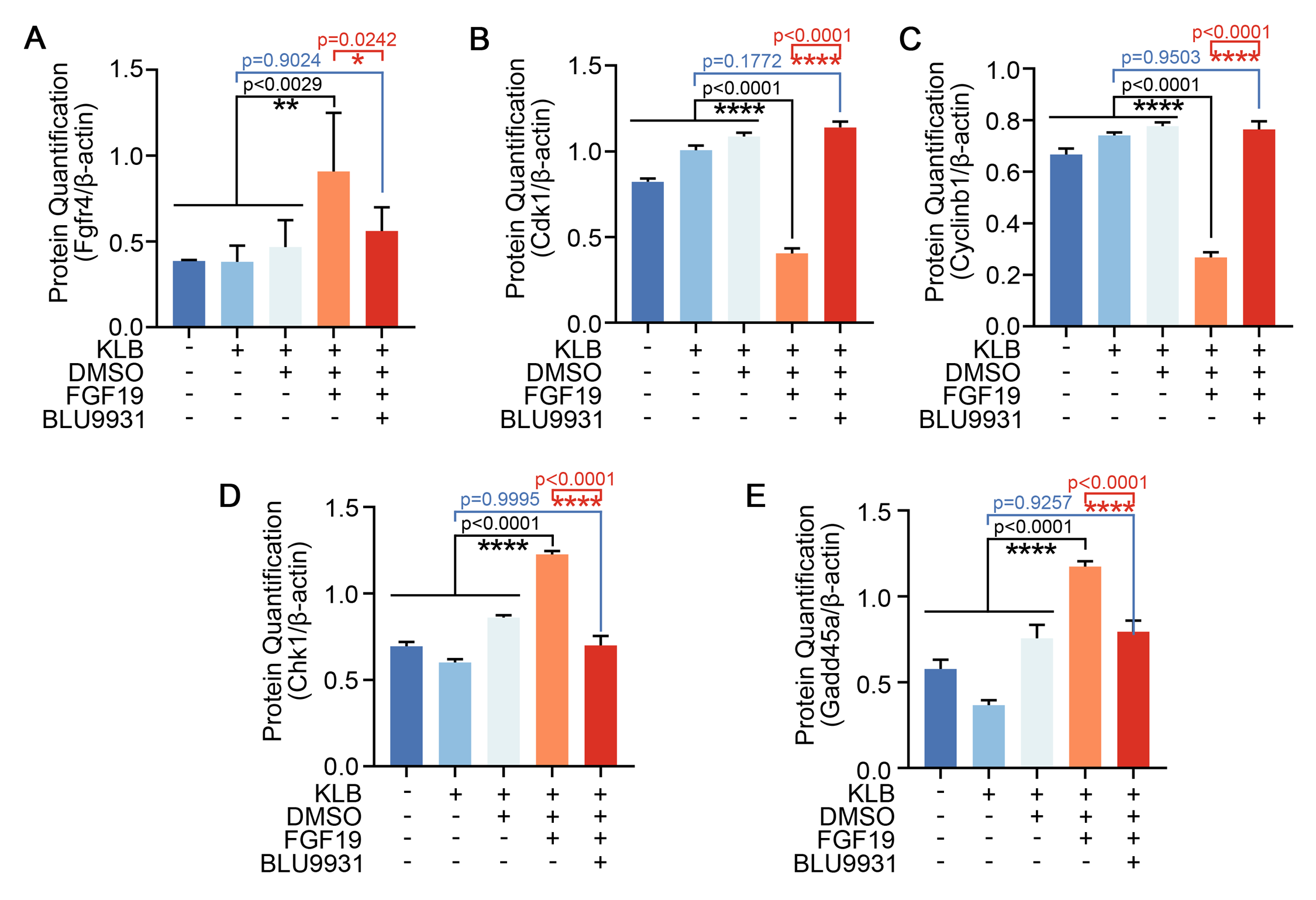
**

**Figure S3. Quantification of cell cycle regulatory proteins in Figure 3(F) in chondrocytes.** All results in **A-E** were obtained from three independent experiments (n = 3). All significance data presented were based on two-tailed Student’s t tests.

**Figure S4**


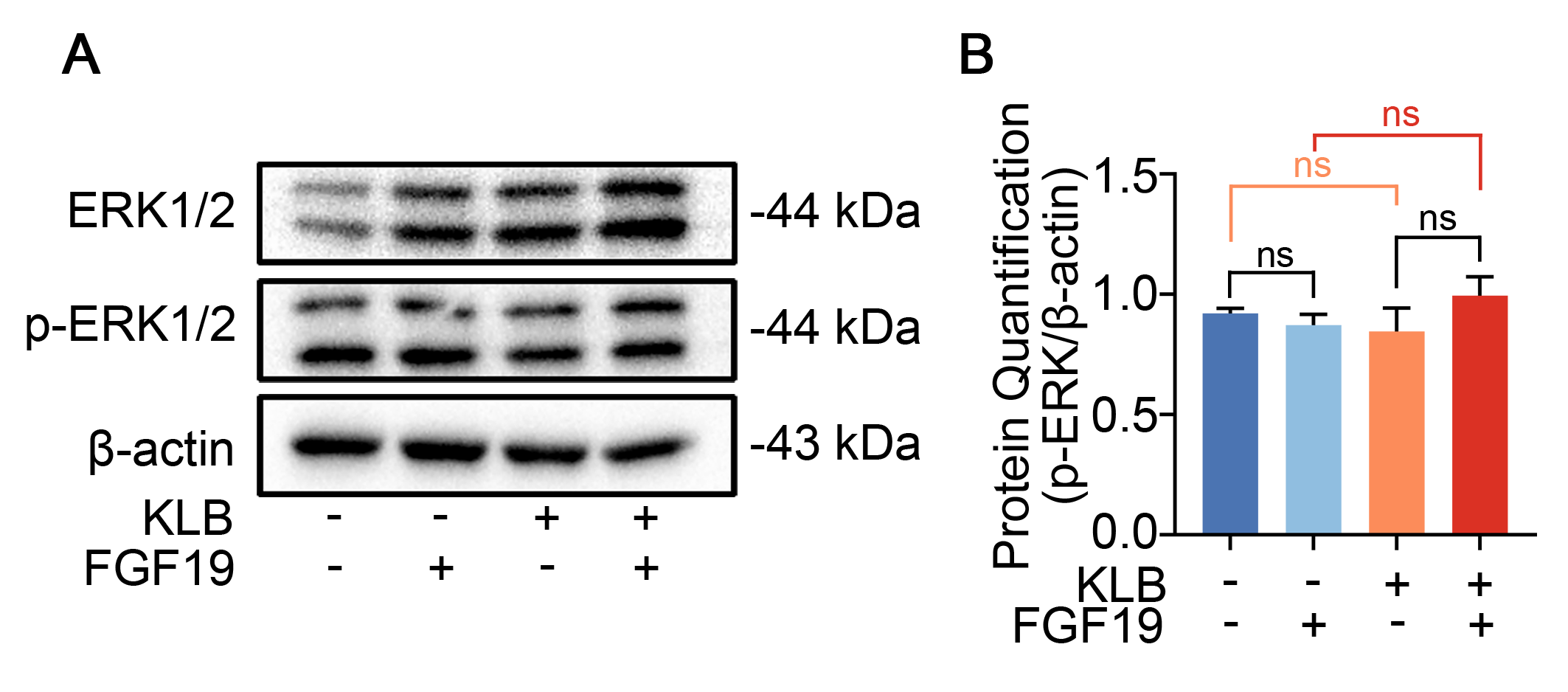


**Figure S4.** T**he expression change of ERK/p-ERK signalling in chondrocytes induced by FGF19.**

**(A)** Representative western blots showing the expression of ERK/p-ERK signalling in chondrocytes induced by 200 ng/ml FGF19 for 72 h in the presence of KLB (200 ng/ml).

**(B)** The graph showing the quantification of ERK/p-ERK protein expression in (A).

Images were chosen based on three independent experiments (n = 3). All significance data presented were based on two-tailed Student’s t tests.

**Figure S5**

**
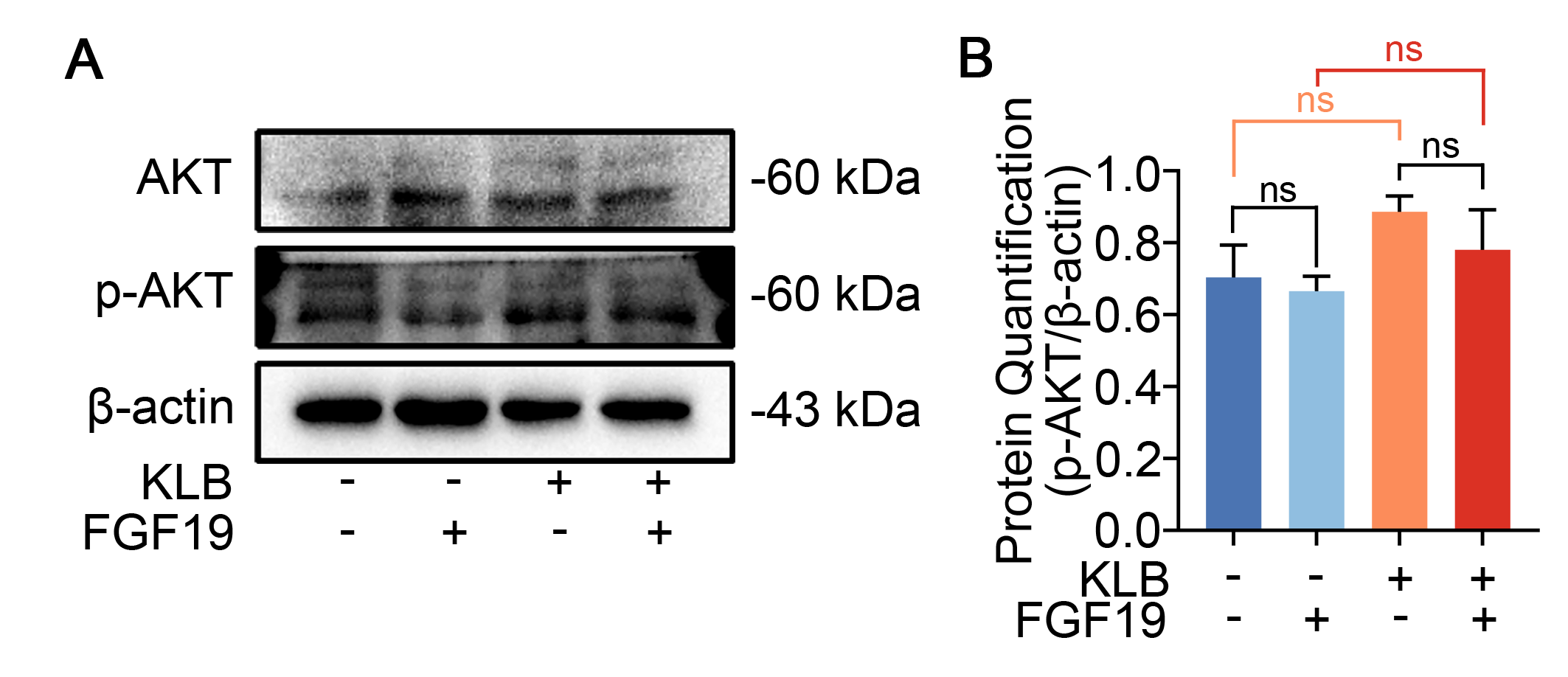
**

**Figure S5. The expression change of AKT/p-AKT signaling in chondrocytes induced by FGF19.**

**(A)** Representative western blots showing the expression of AKT/p-AKT signalling in chondrocytes induced by 200 ng/ml FGF19 for 72 h in the presence of KLB (200 ng/ml).

**(B)** The graph showing the quantification of AKT/p-AKT in (A).

Images were chosen based on three independent experiments (n = 3). All significance data presented were based on two-tailed Student’s t tests.

**Figure S6**

**
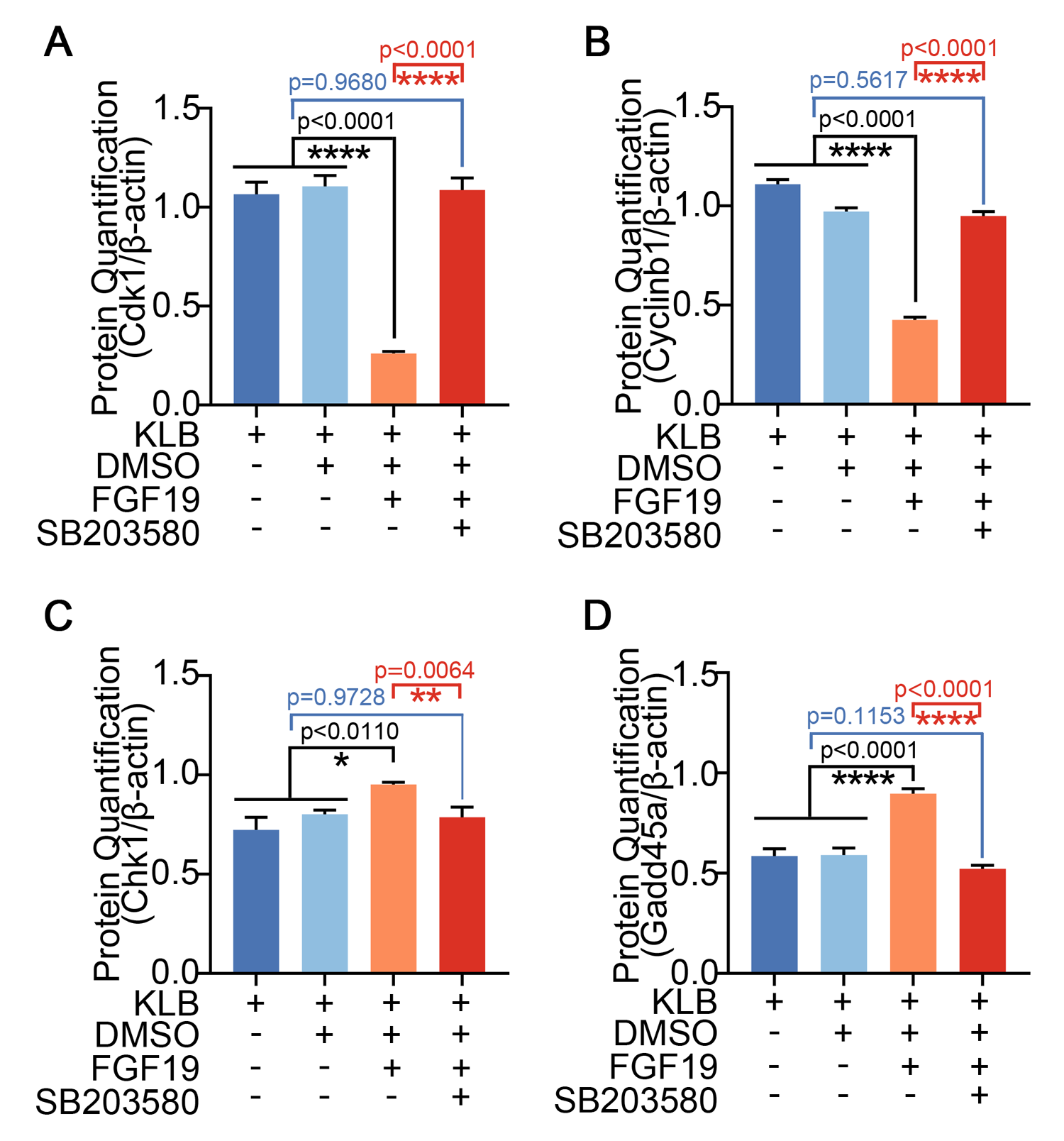
**

**Figure S6.** Quantification of cell cycle regulatory proteins expression in **Figure 5(D)** in chondrocytes. The results were chosen based on three independent experiments (n = 3). All significance data presented were based on two-tailed Student’s t tests.
