# Supplementary material for: FGF19 induces the cell cycle arrest at G2-phase in chondrocytes
Source: Cell Death Discov. 2023 Jul 15;9:250. doi: 10.1038/s41420-023-01543-6 (PMC10349815; doi:10.1038/s41420-023-01543-6)
Supplement: Supplementary file 2 — Original data files [file 41420_2023_1543_MOESM2_ESM.docx]

Supplemental Material- Original key data in the study

1. **The original western blots for Figure 2A**

**
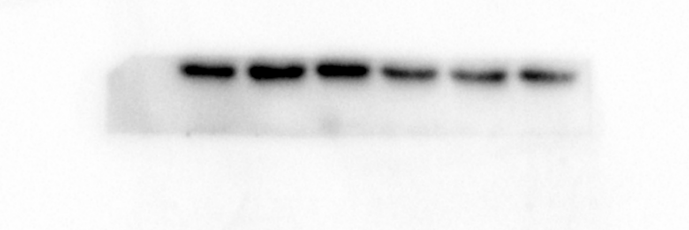
Cdk1 Cyclinb1**

**Cdk1**

**
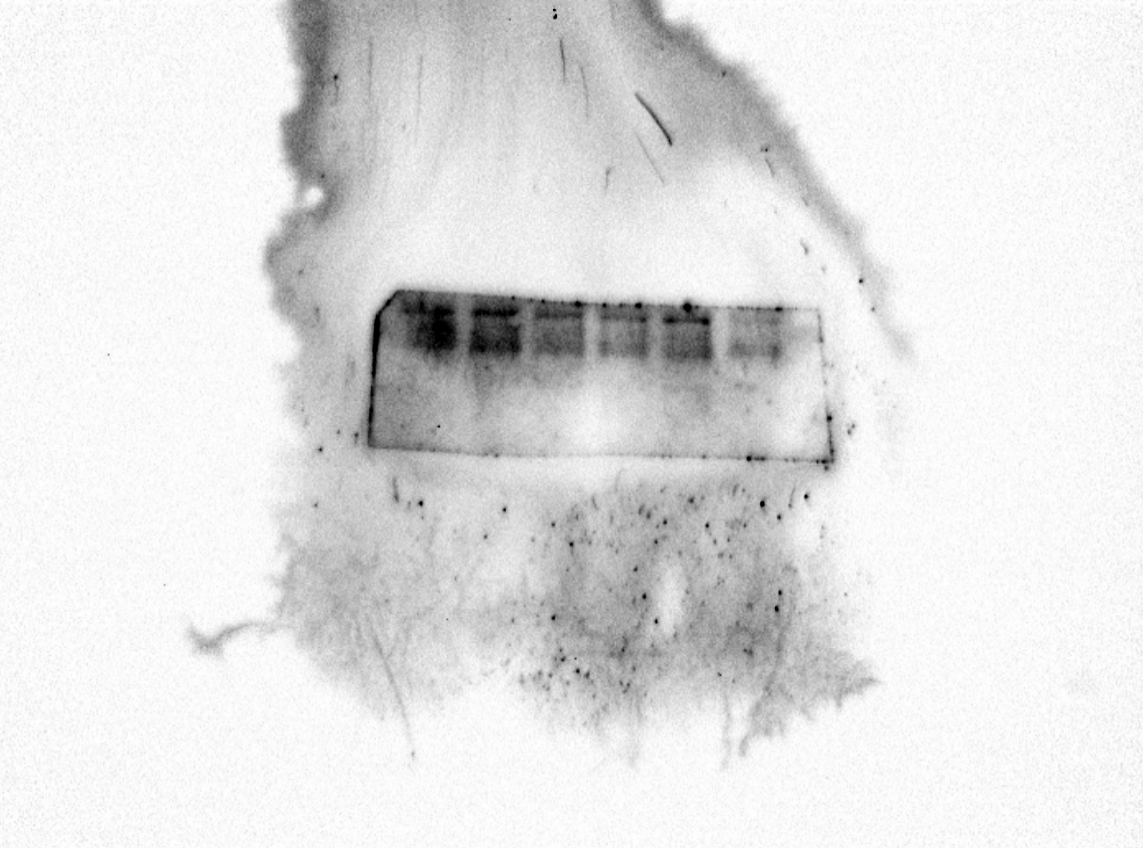
**

**55 kDa**

**Cyclinb1**

**34 kDa**


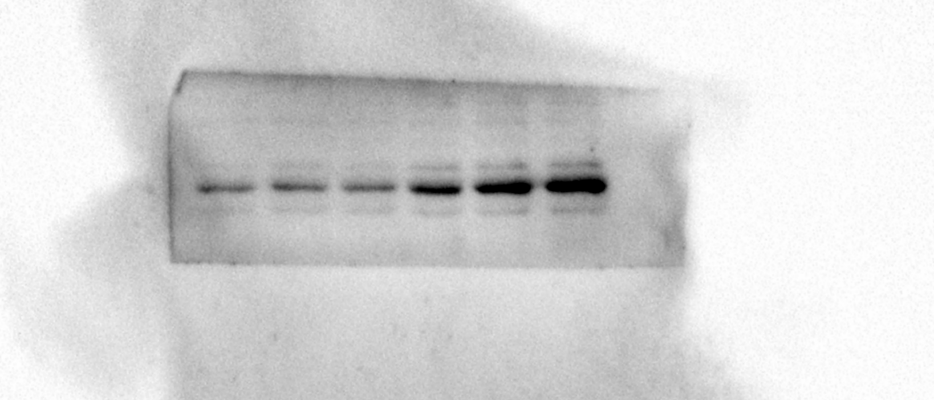
**Chk1 Gadd45a**

**
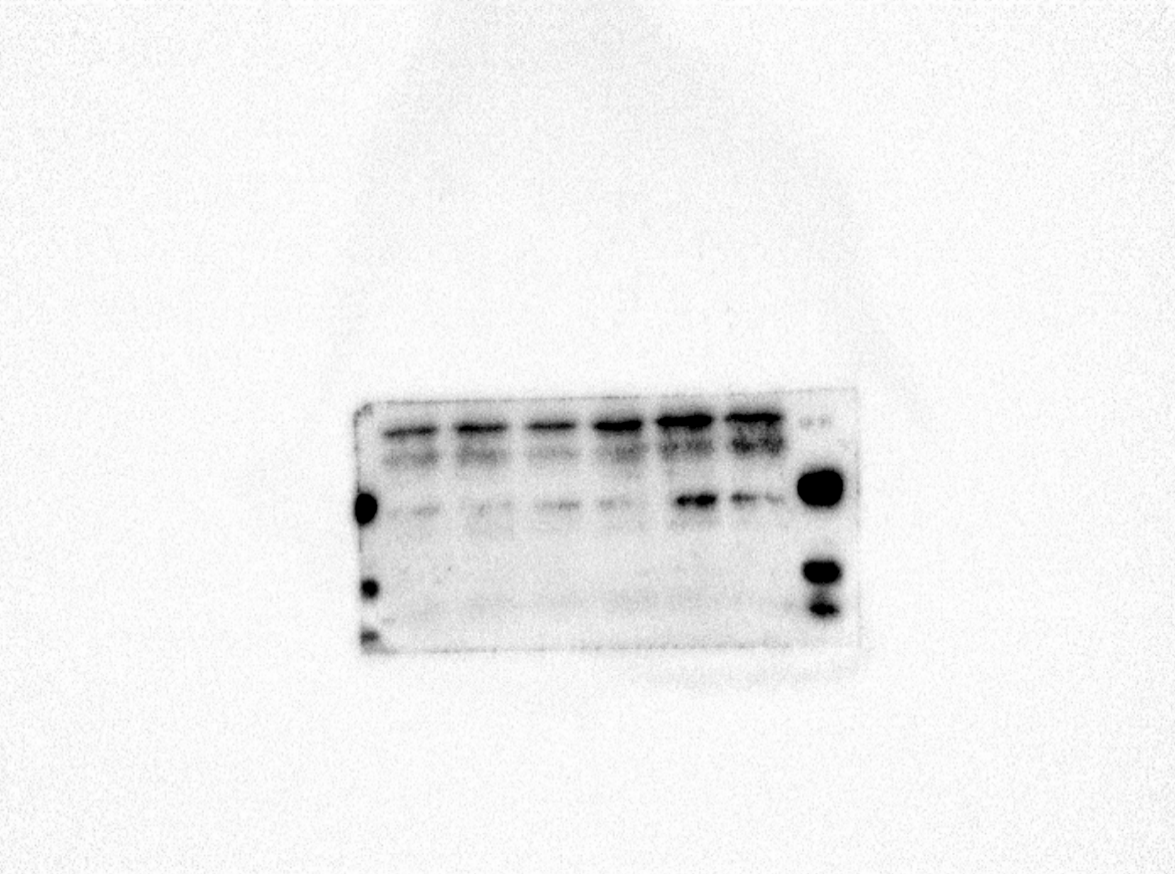
**

**Gadd45a**

**18 kDa**

**54 kDa**

**Chk1**

**
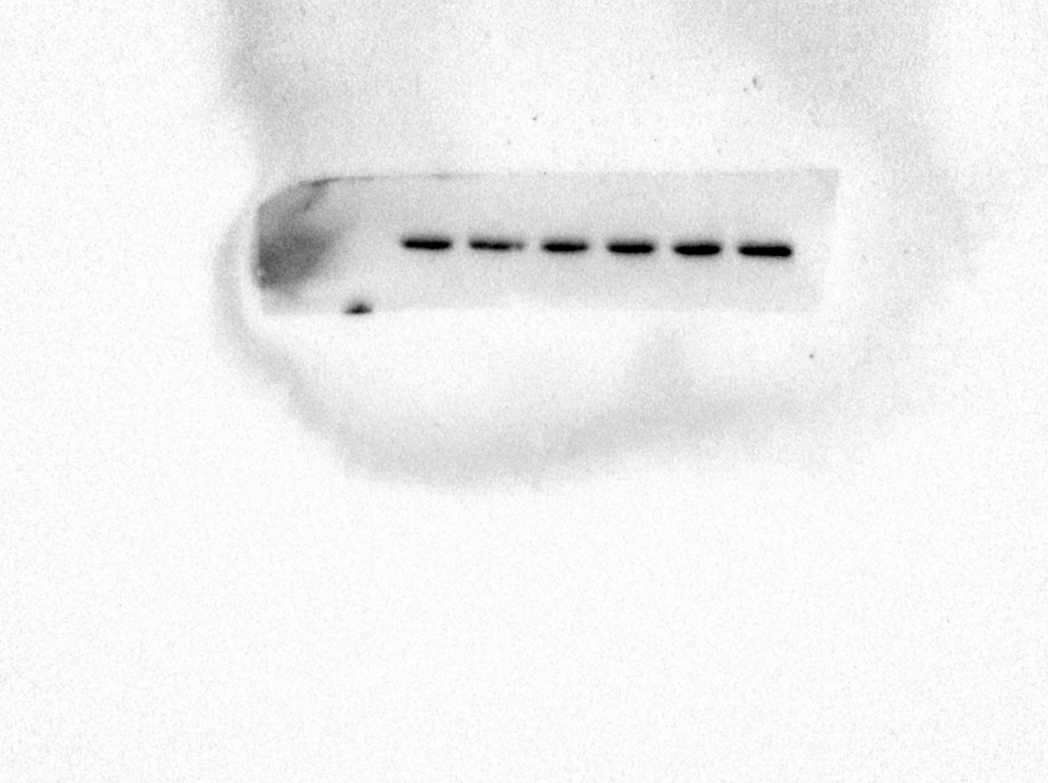
β-actin**

**β-actin**

**43 kDa**

1. **The original western blots for Figure 3B**

**
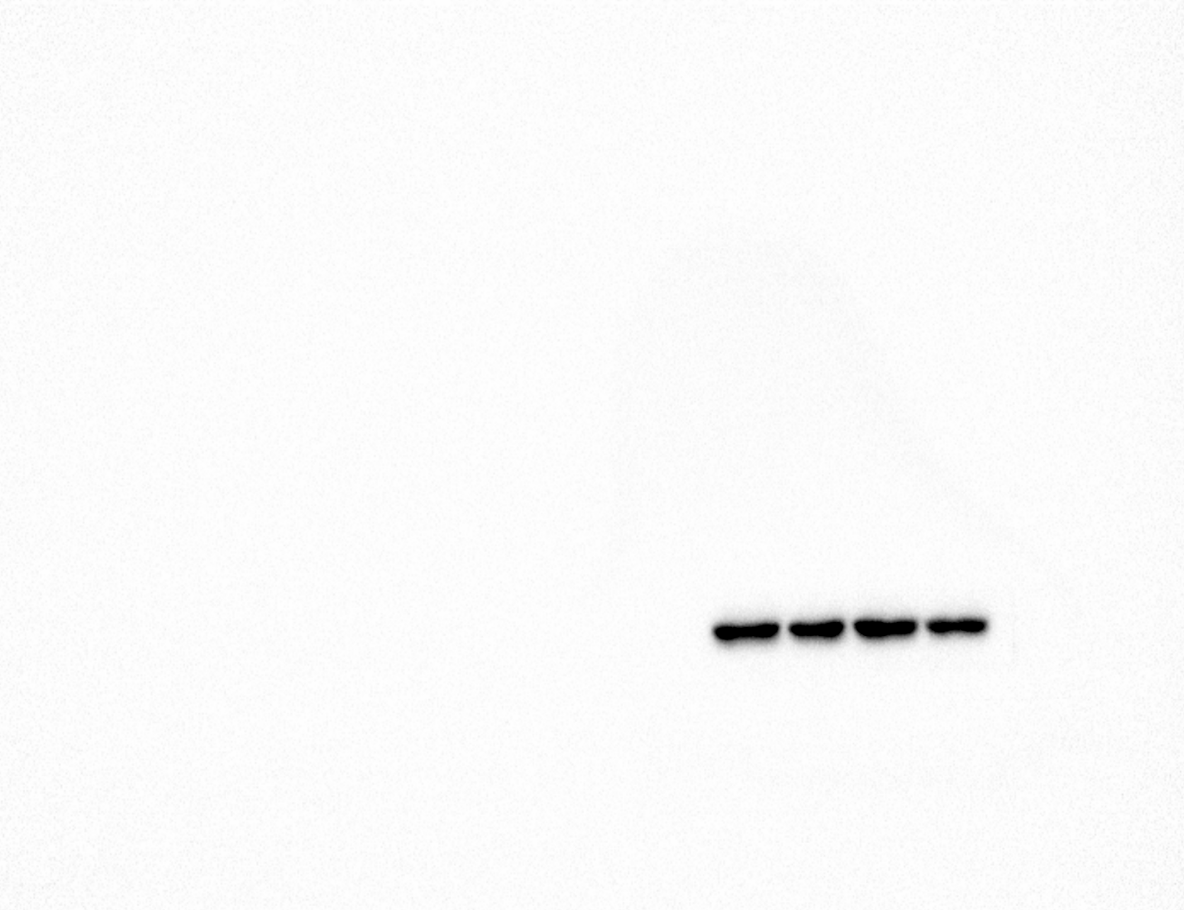
FGFR4 β-actin**


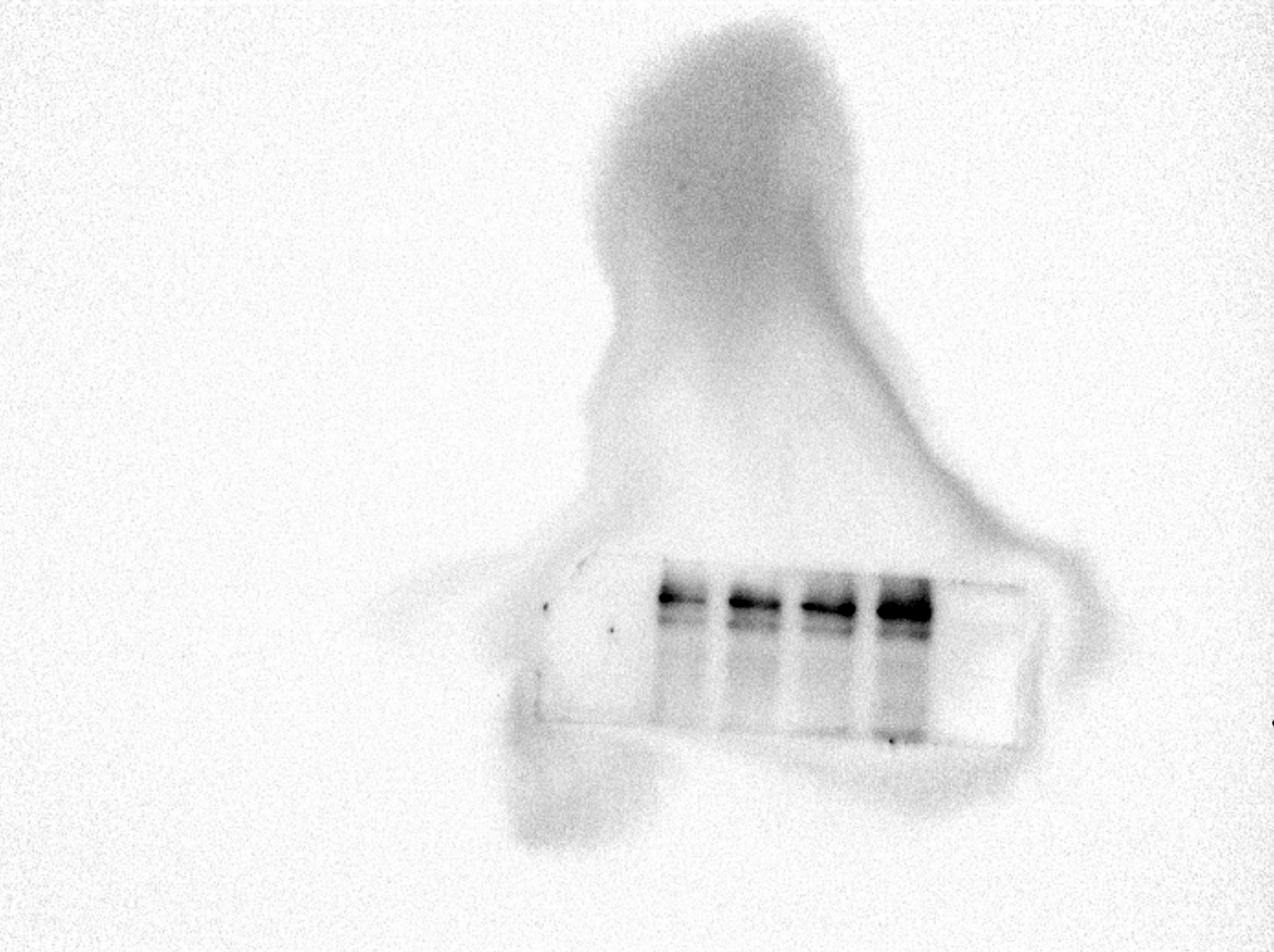


**43 kDa**

**β-actin**

**88 kDa**

**FGFR4**

1. **The original western blots for Figure 3F**

**
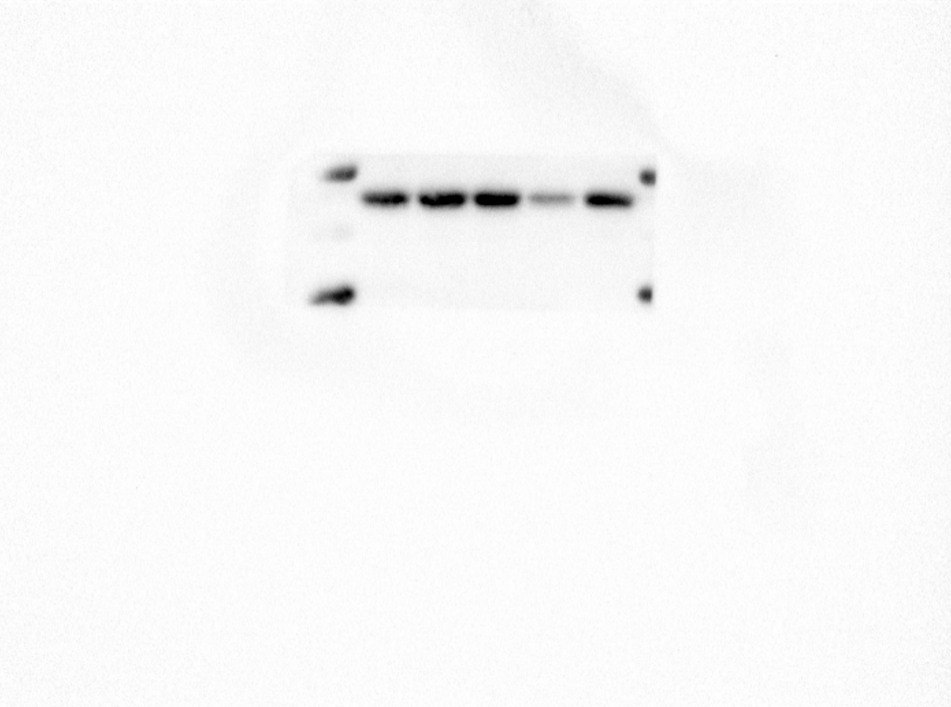
**
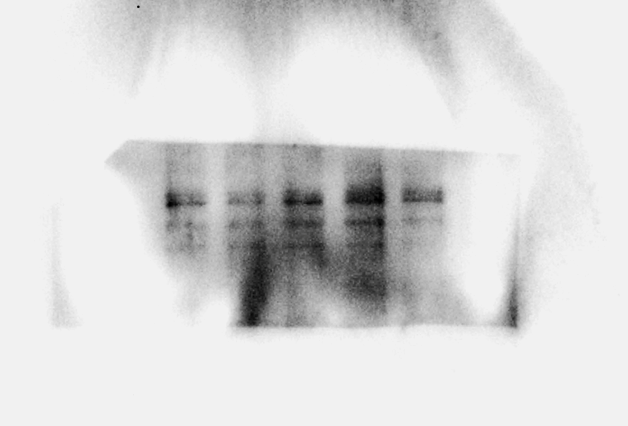
**FGFR4 Cdk1**

**FGFR4**

**34 kDa**

**88 kDa**

**Cdk1**

**
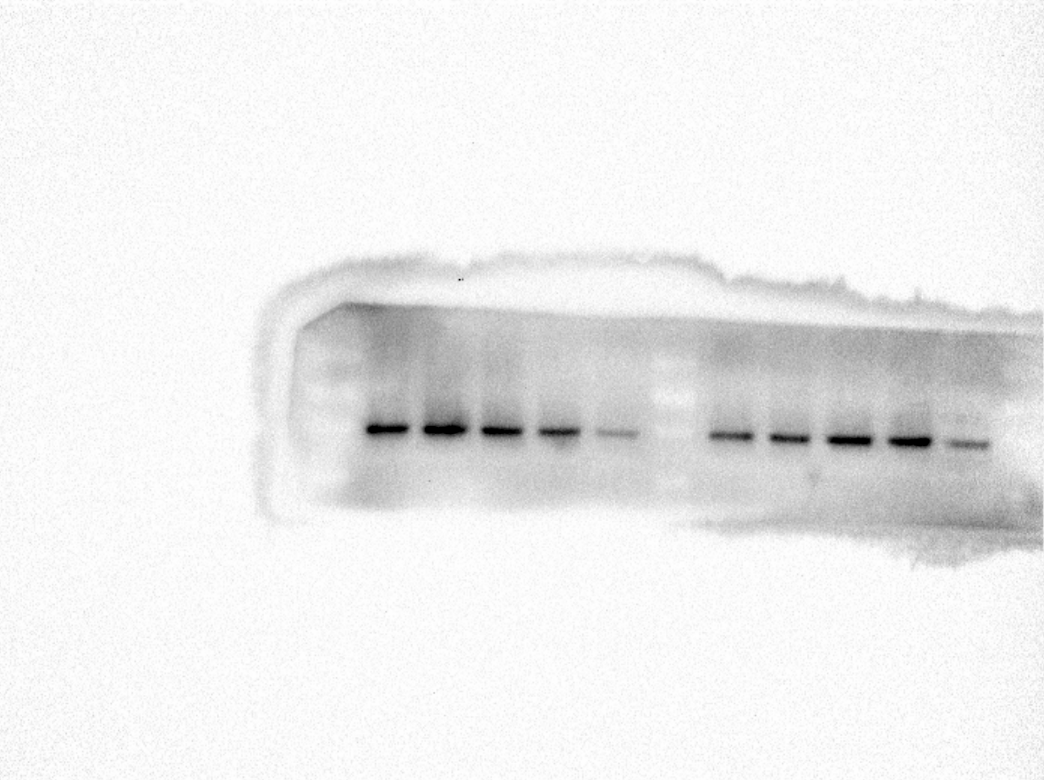

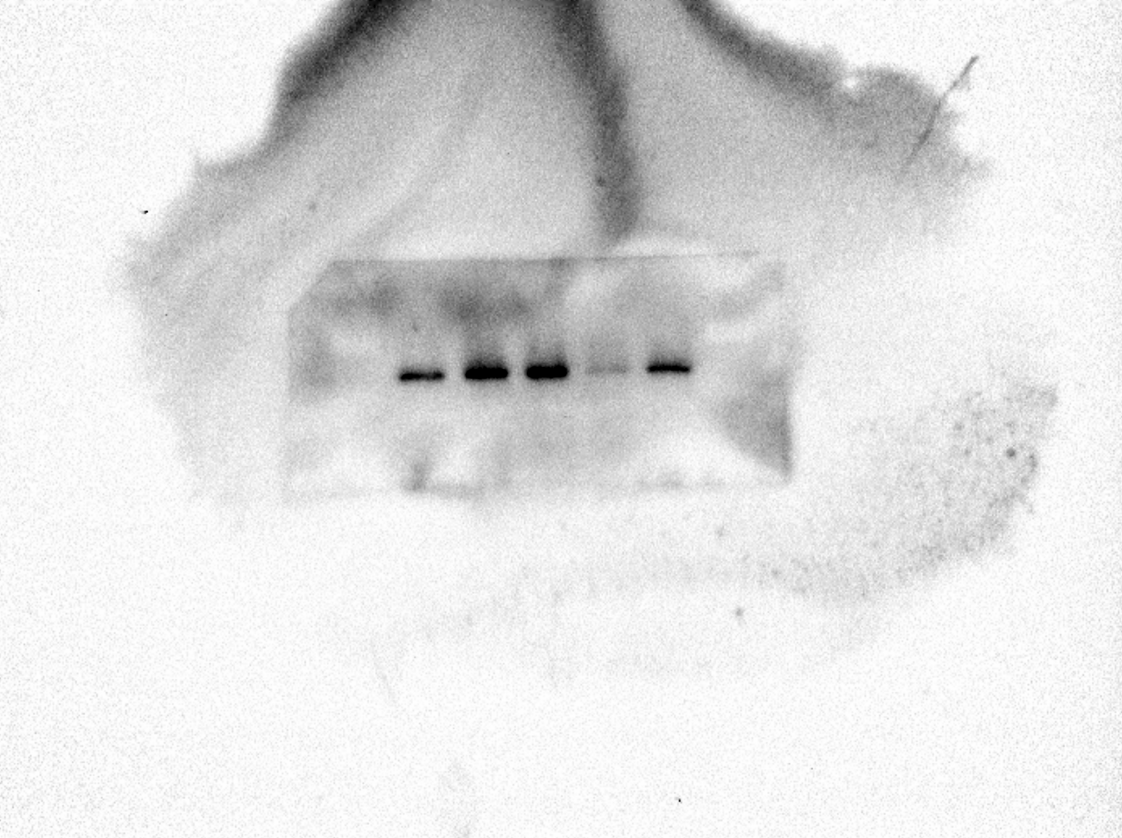
Cyclinb1 Chk1**

**54 kDa**

**55 kDa**

**Cyclinb1**

**Chk1**

**
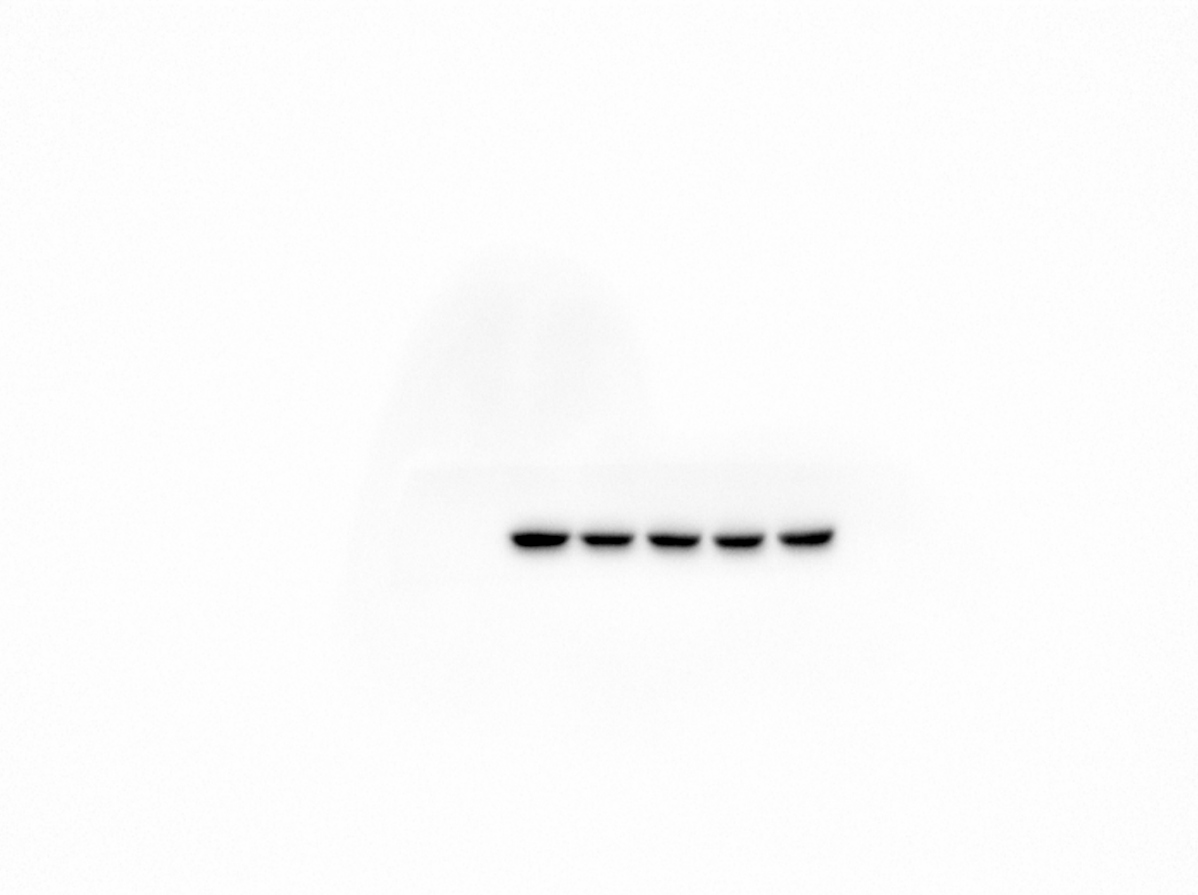

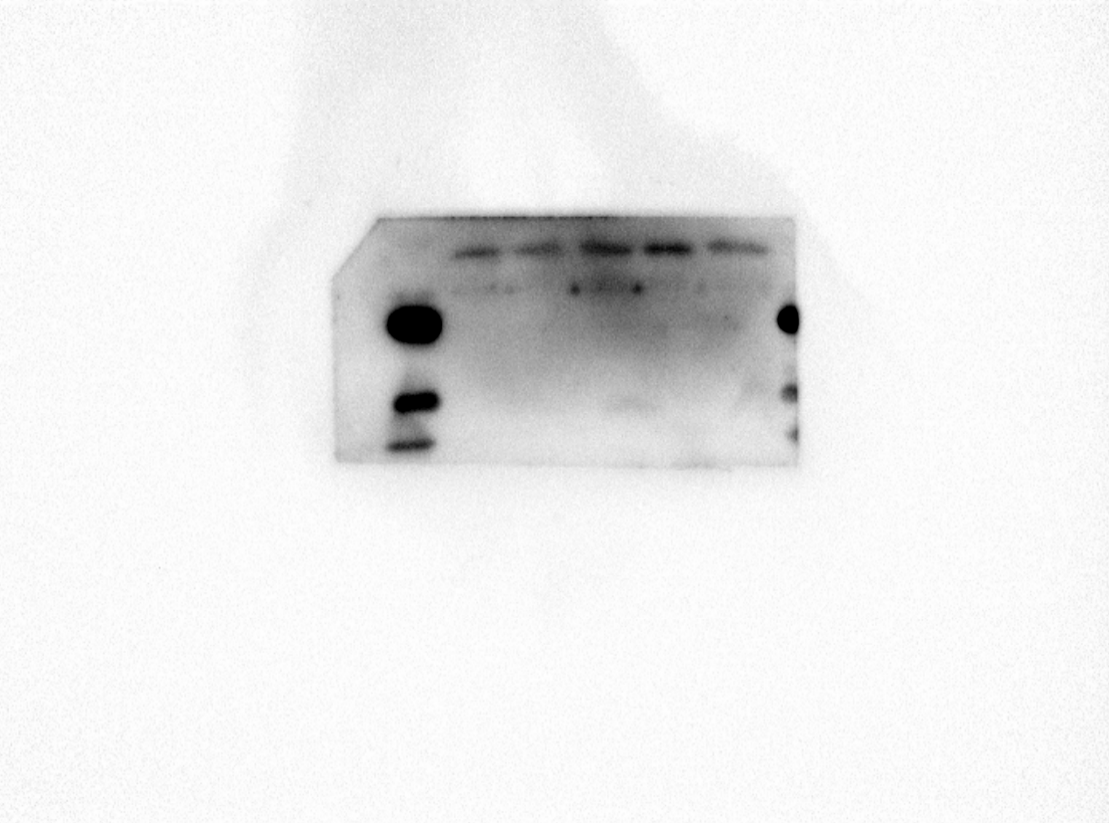
Gadd45a β-actin**

**43 kDa**

**β-actin**

**18 kDa**

**Gadd45a**

1. **The original western blots for Figure 4A**

**
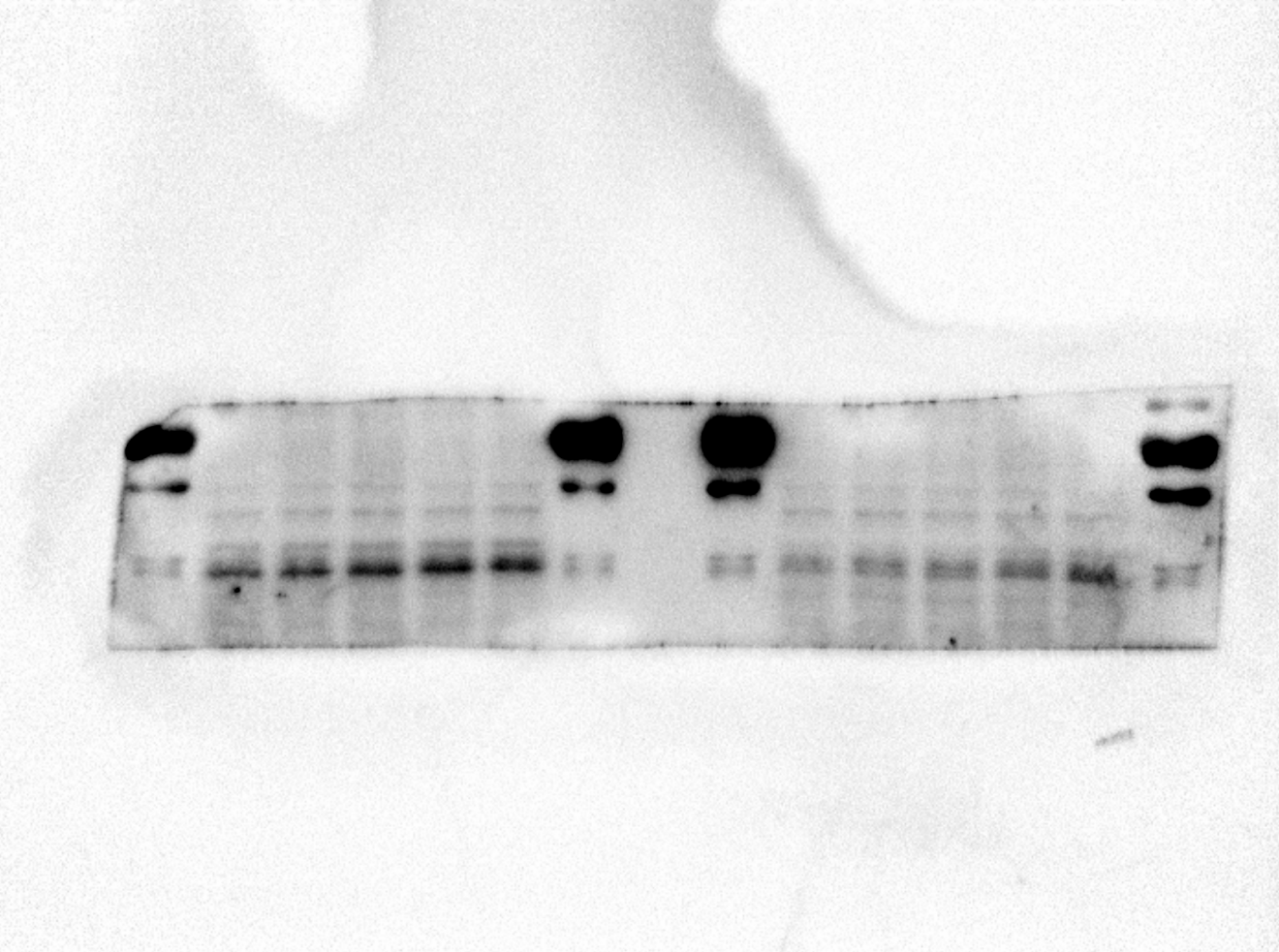
p38 p-p38**

**
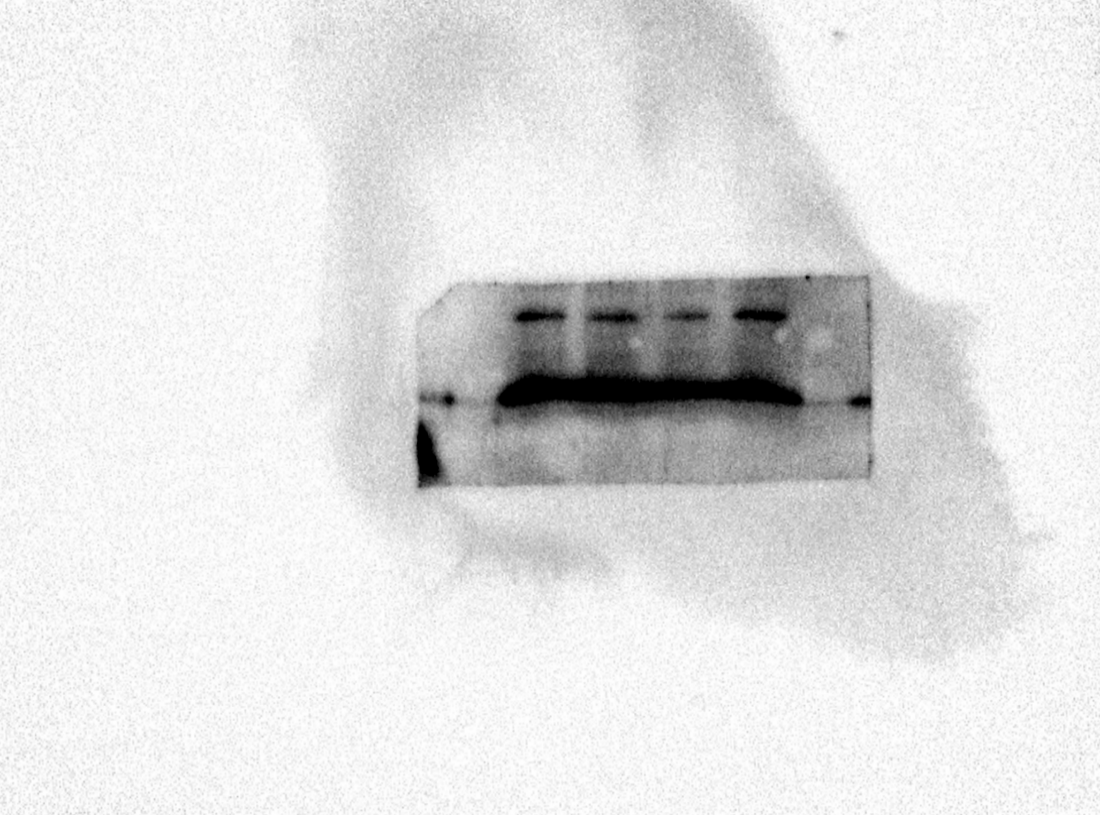
**

**41 kDa**

**p38**

**43 kDa**

**p-p38**

**
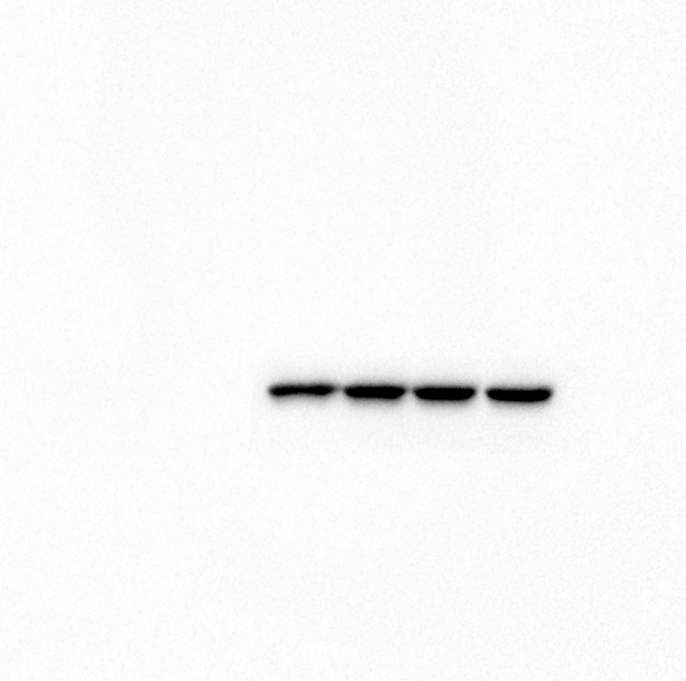
β-actin**

**43 kDa**

**β-actin**

1. **The original western blots for Figure 4D**


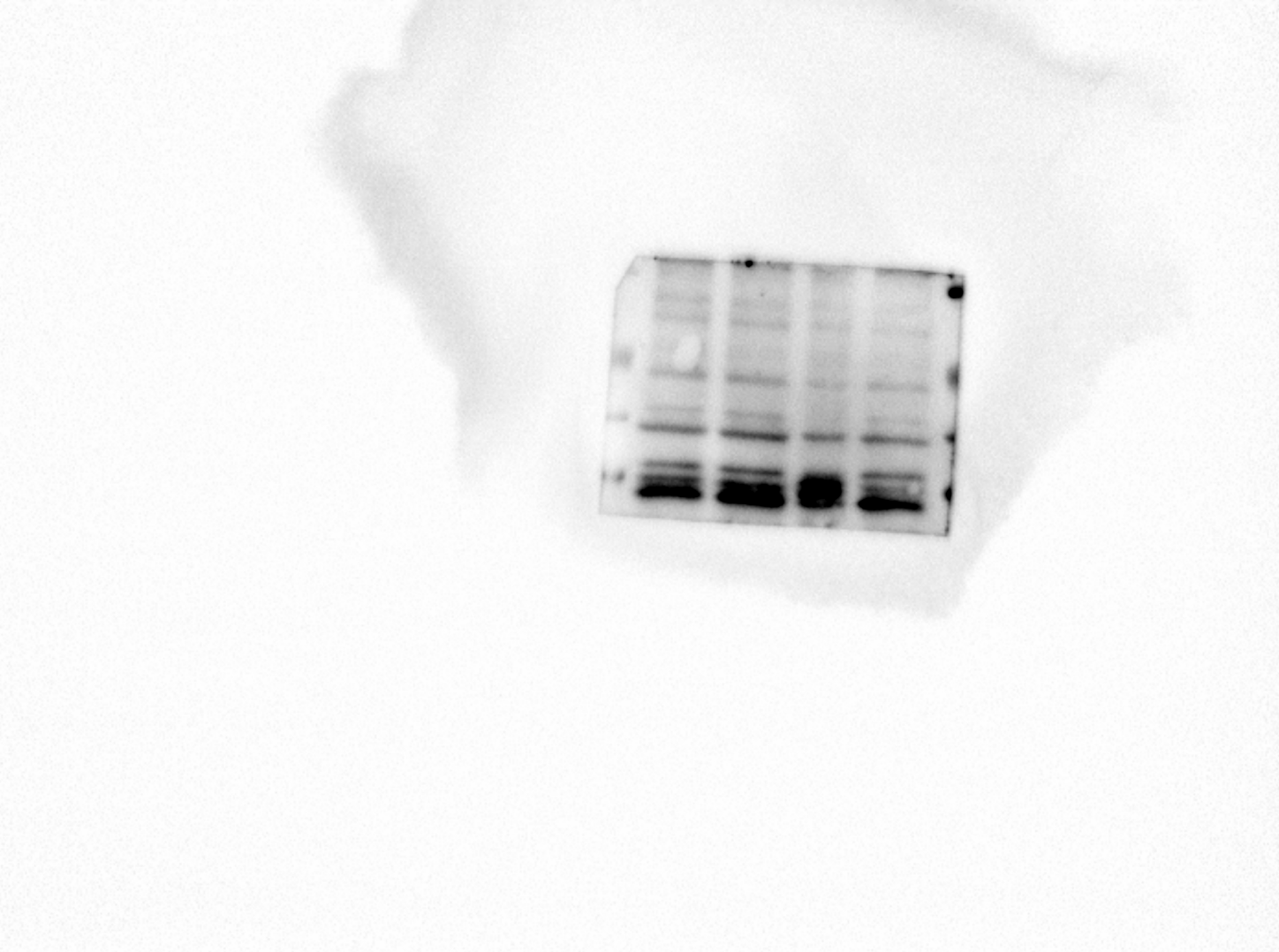
**
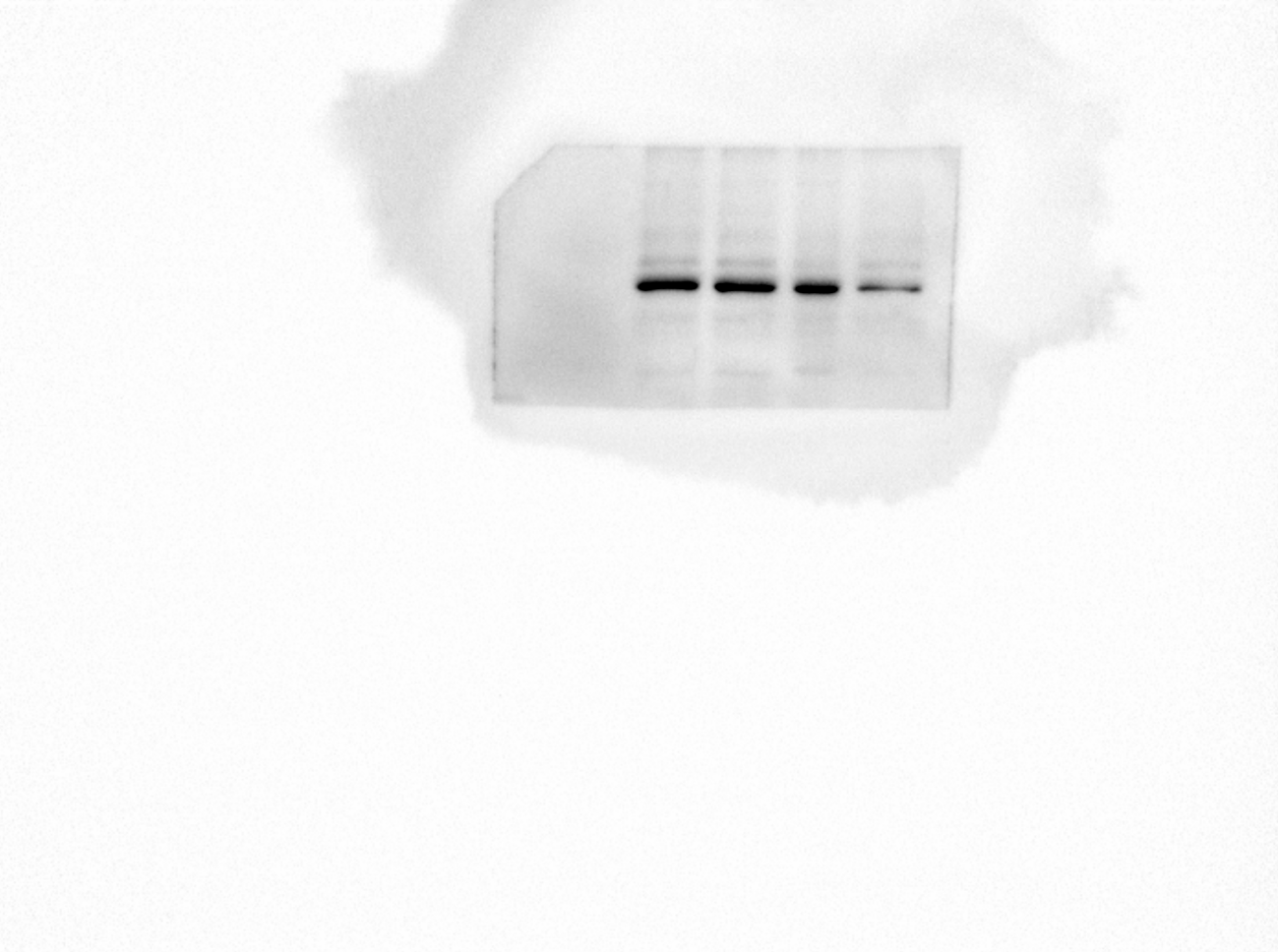
p38 p-p38**

**41 kDa**

**p38**

**43 kDa**

**p-p38**

**
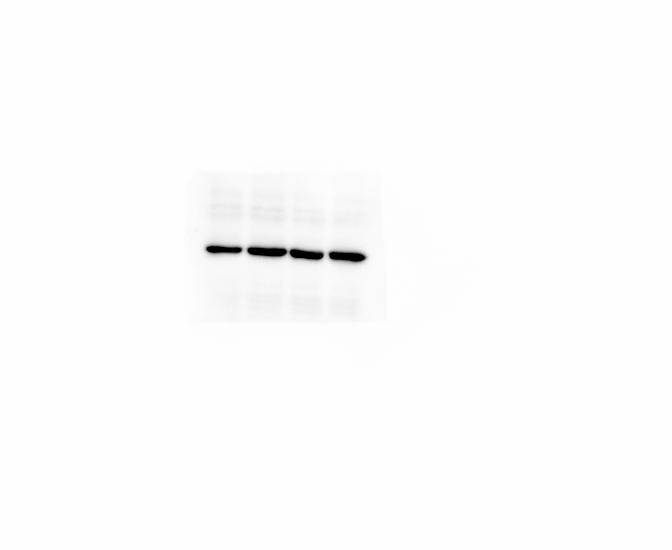
β-actin**

**β-actin**

**43 kDa**

1. **The original western blots for Figure 5A**


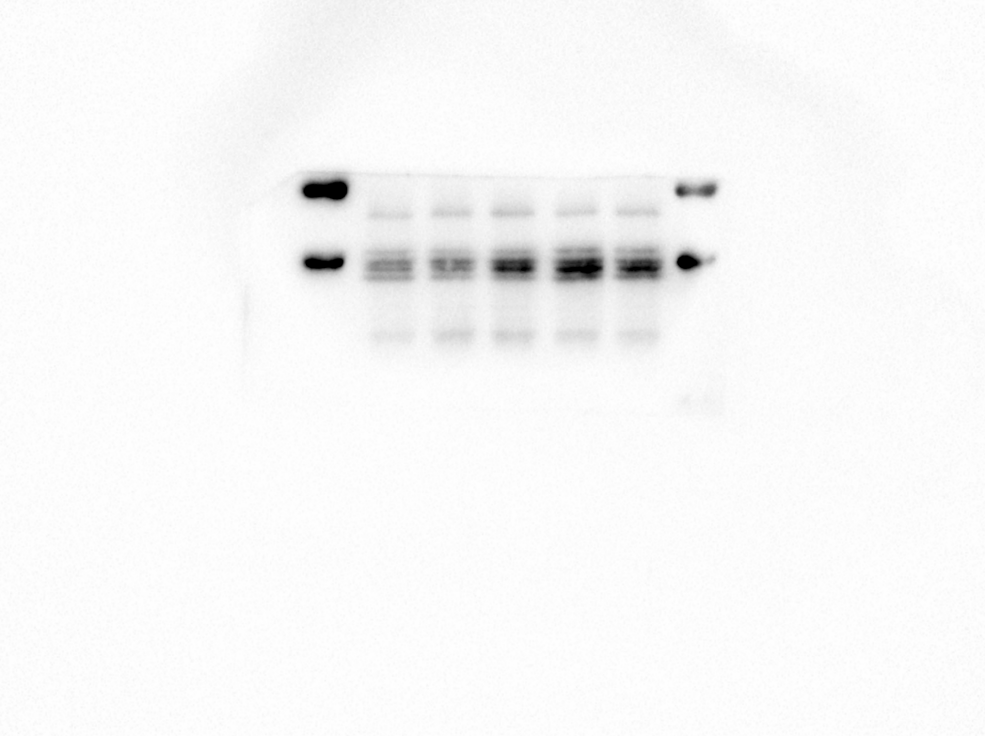

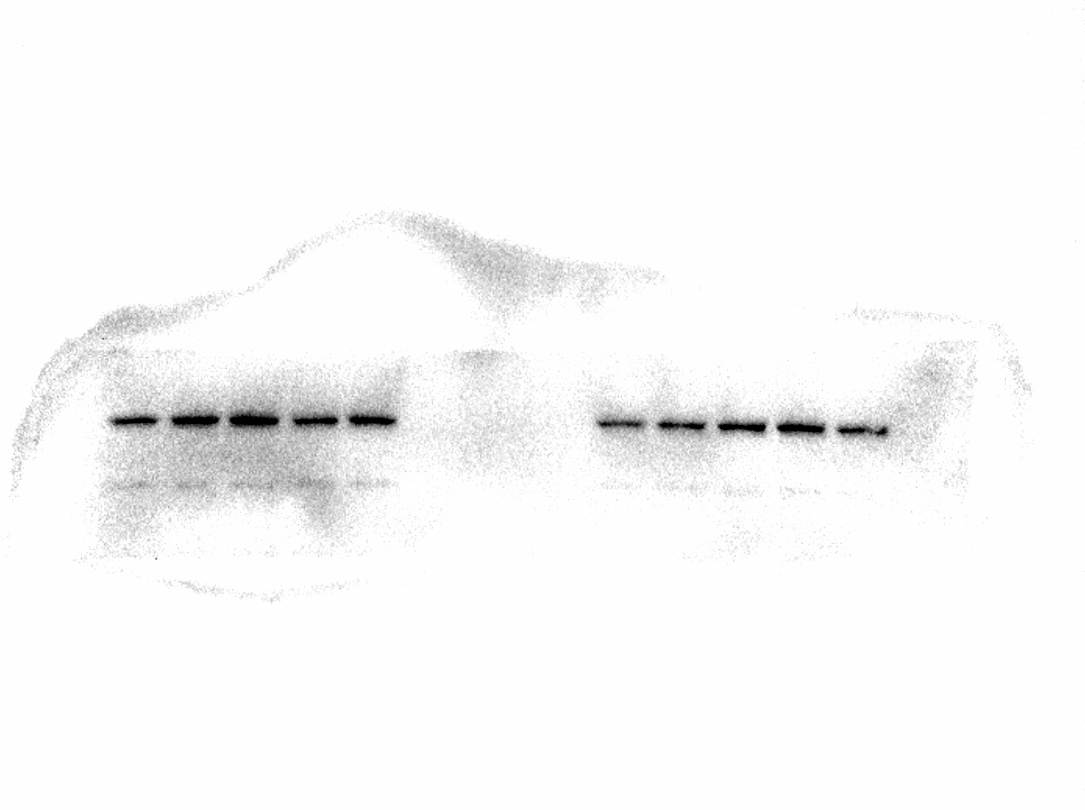
**p38 p-p38**

**p38**

**43 kDa**

**p-p38**

**41 kDa**

**
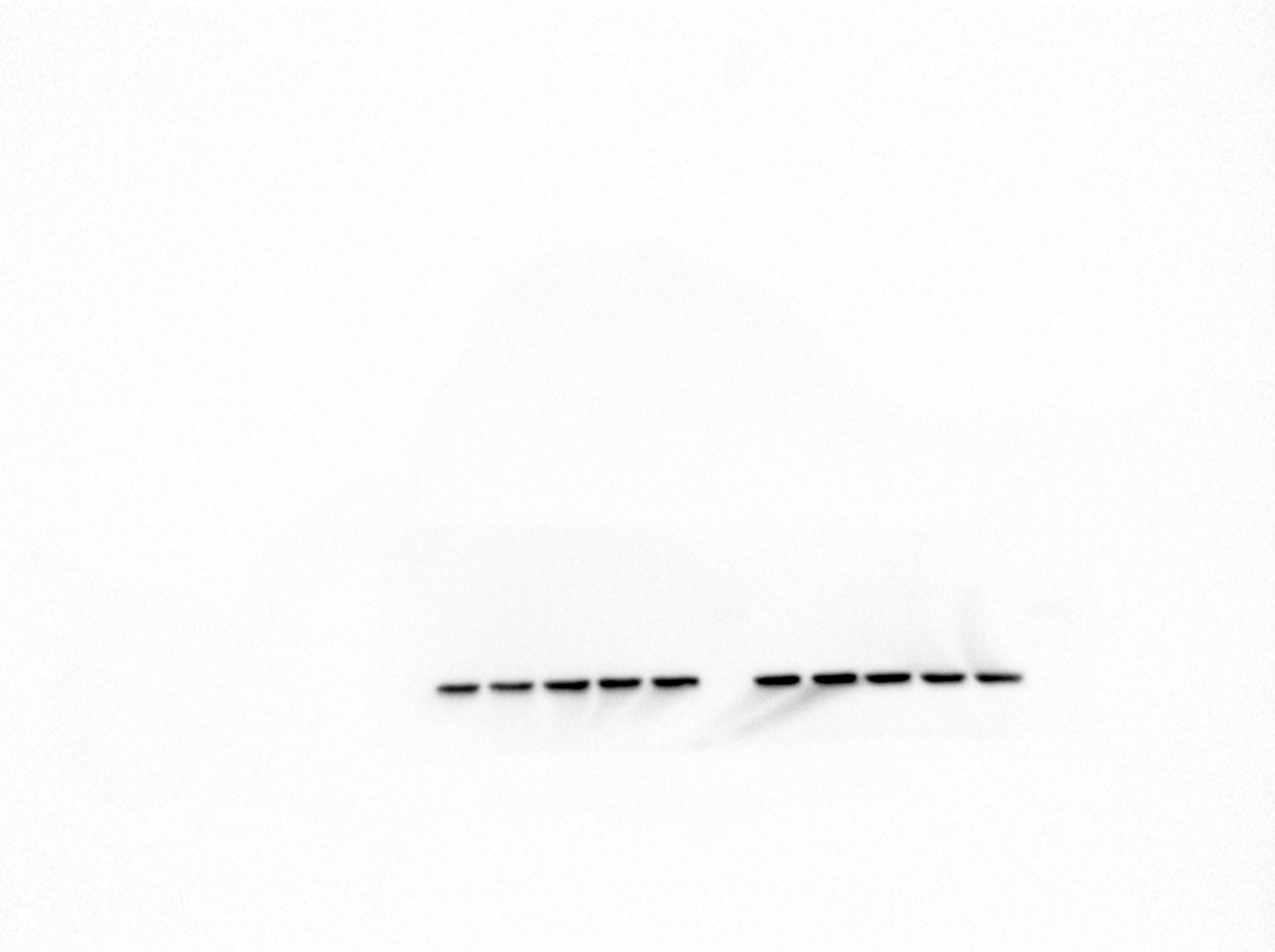
β-actin**

**43 kDa**

**β-actin**

1. **The original western blots for Figure 5D**

**
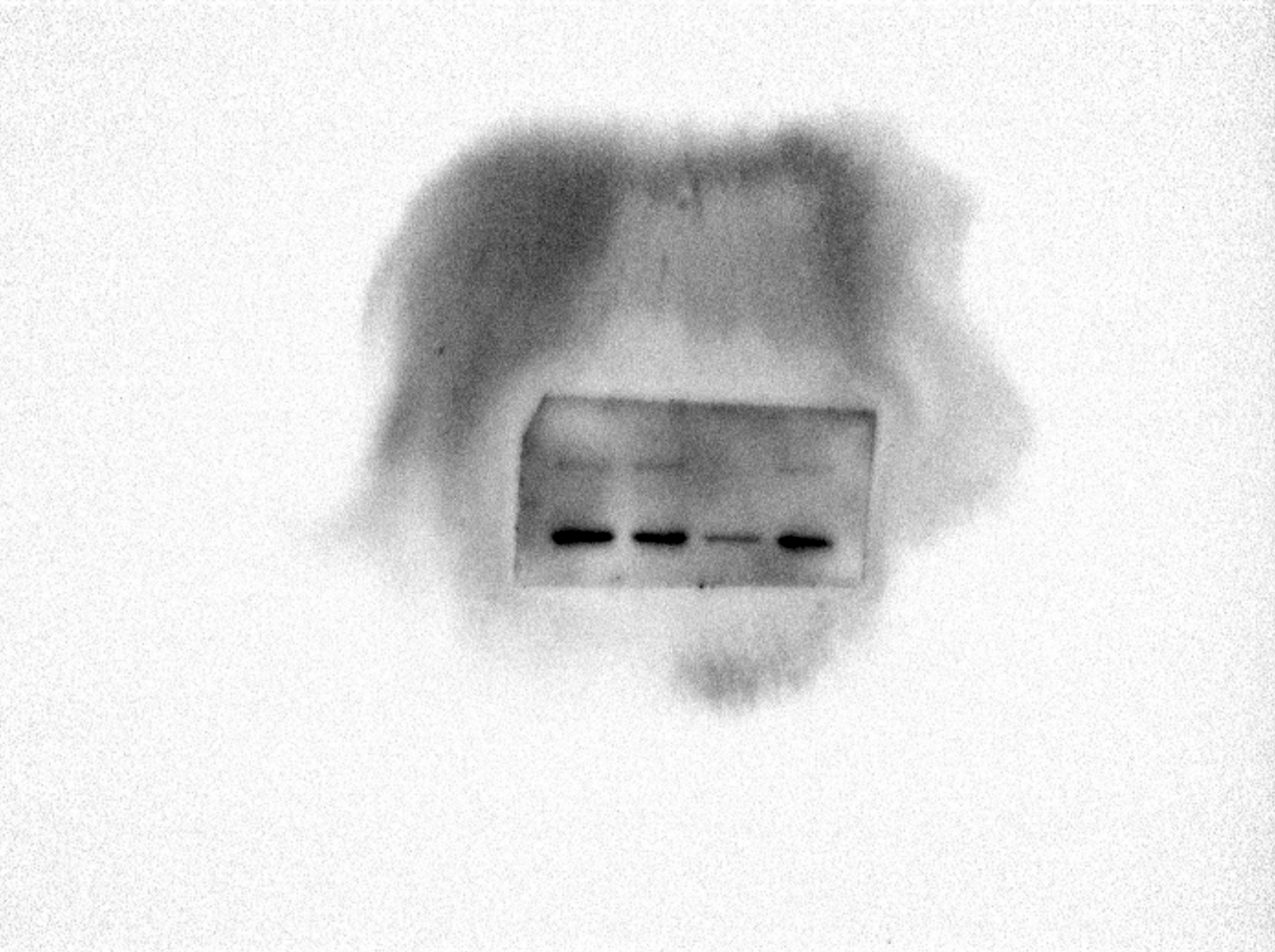

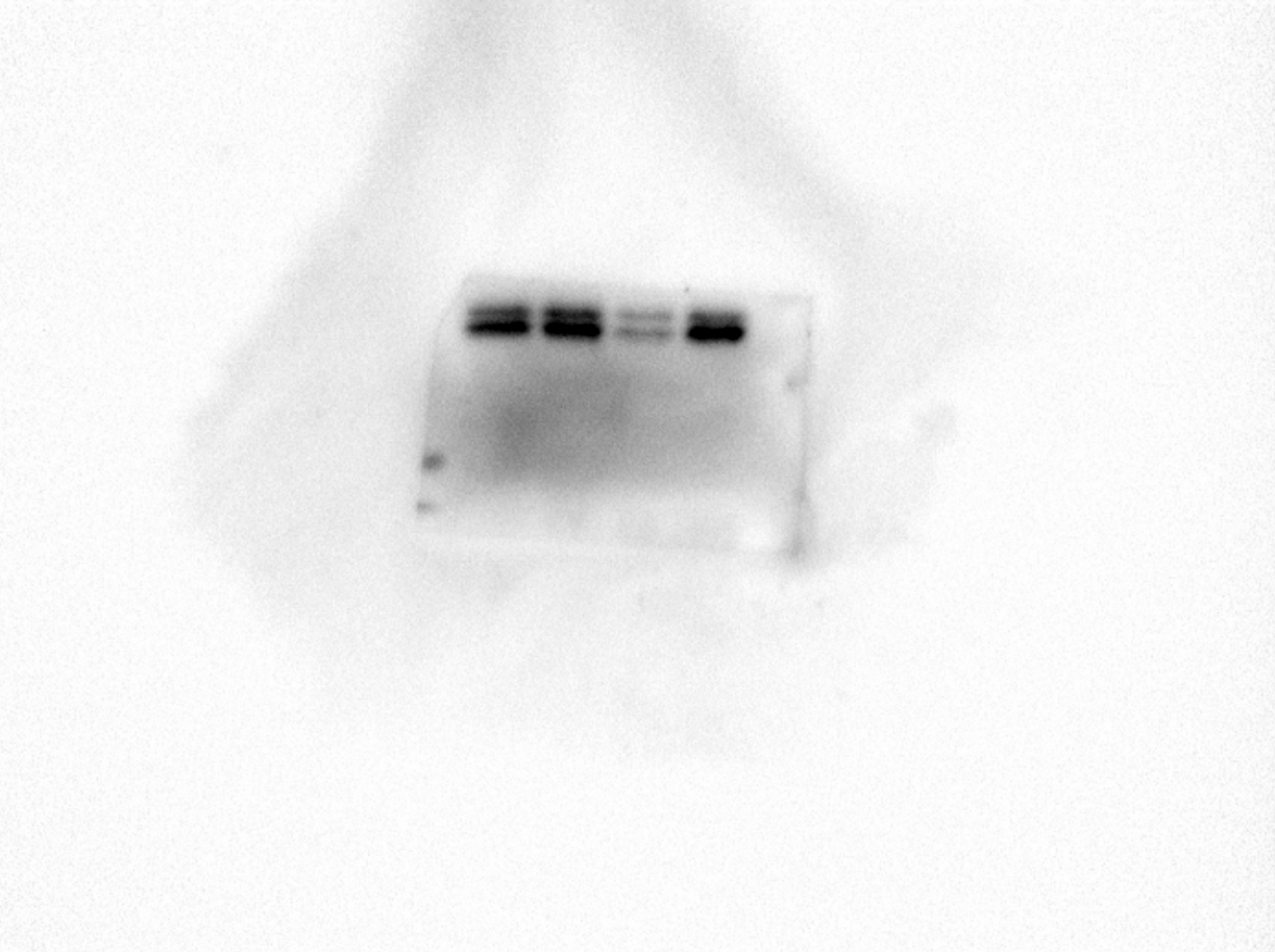
Cdk1 Cyclinb1**

**Cdk1**

**34 kDa**

**55 kDa**

**Cyclinb1**

**Chk1 Gadd45a**

**
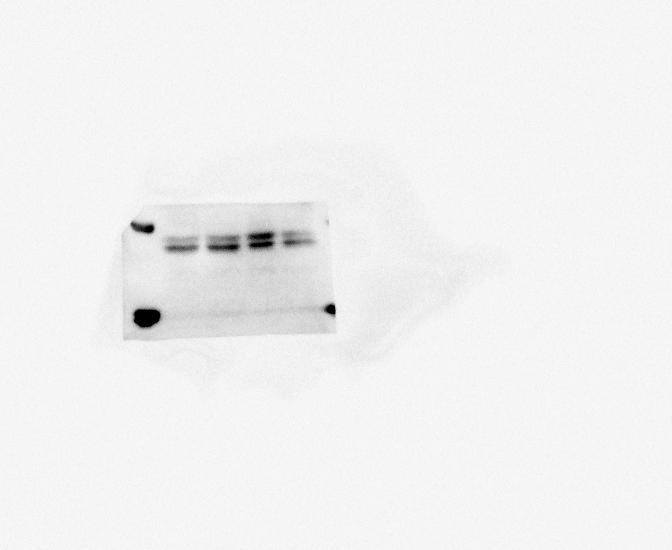
**
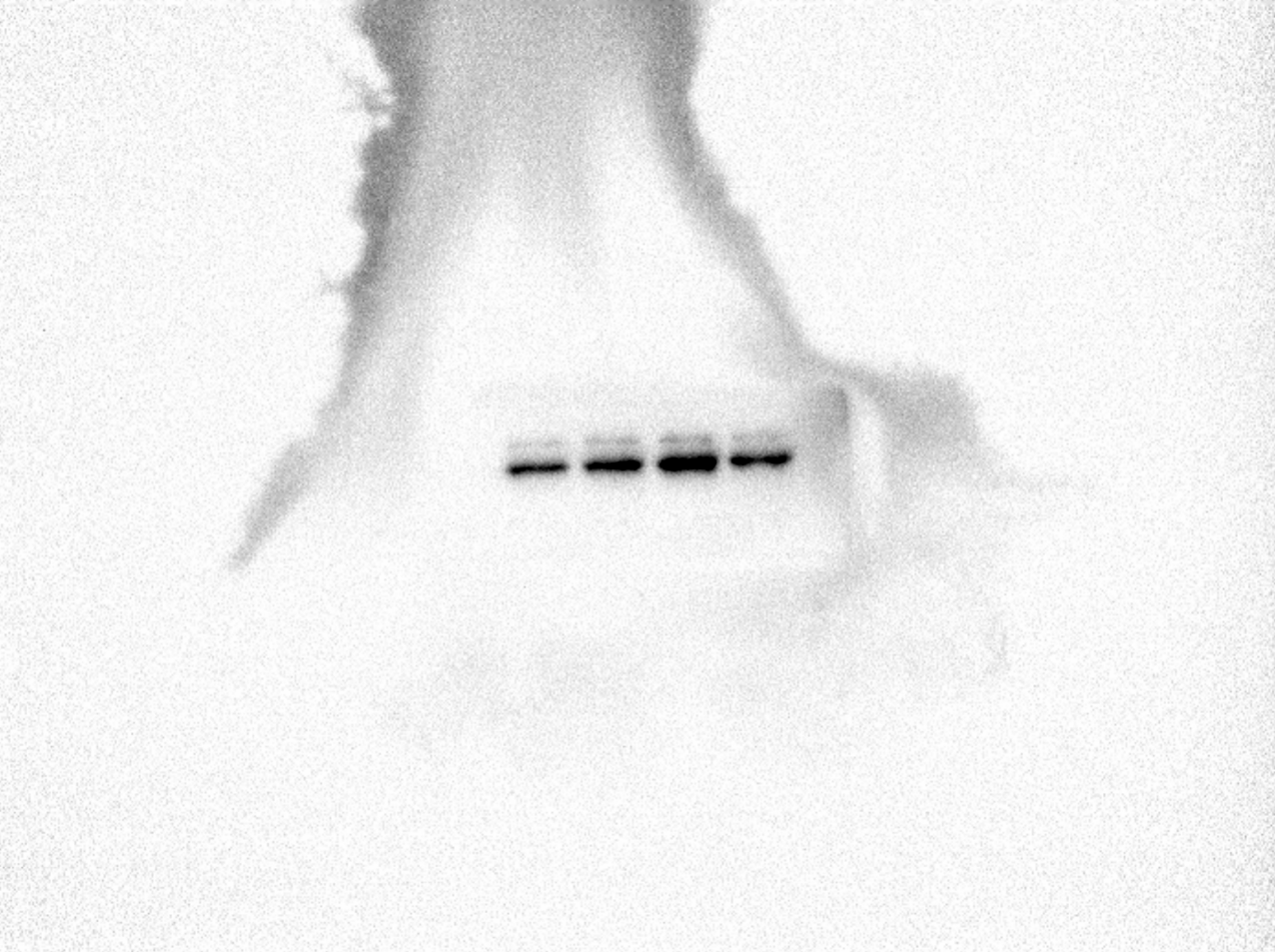


**18 kDa**

**Gadd45a**

**54 kDa**

**Chk1**

**
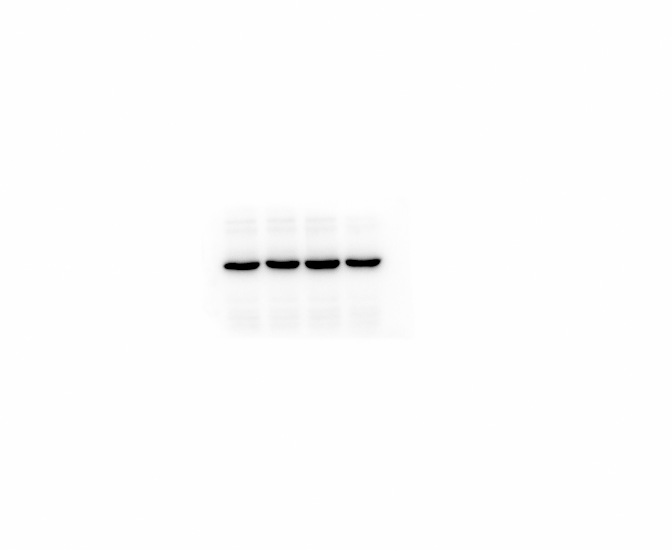
β-actin**

**β-actin**

**43 kDa**
